# Molecular epidemiology of carbapenem-resistant *Acinetobacter baumannii* group in Taiwan

**DOI:** 10.1128/msphere.00793-24

**Published:** 2024-12-31

**Authors:** Tran Lam Tu Quyen, Yu-Chia Hsieh, Shiao-Wen Li, Lii-Tzu Wu, Ya-Zhu Liu, Yi-Jiun Pan

**Affiliations:** 1 Department of Biological Science and Technology, College of Life Science, China Medical University, Taichung, Taiwan; 2 Department of Microbiology and Immunology, School of Medicine, College of Medicine, China Medical University, Taichung, Taiwan; 3 Department of Pediatrics, Chang Gung Children’s Hospital, Chang Gung Memorial Hospital, Chang Gung University, College of Medicine, Taoyuan, Taiwan; 4 Department of Life Sciences, National University of Kaohsiung 63286 , Kaohsiung, Taiwan; University of Nebraska Medical Center College of Medicine, Omaha, Nebraska, USA

**Keywords:** capsular types, *wzy *multiplex PCR, *Acinetobacter baumannii *group, carbapenemase genes

## Abstract

*Acinetobacter,* particularly the *Acinetobacter baumannii* group, is a major cause of nosocomial infections, and carbapenem-resistant *Acinetobacter* spp. are important human pathogens. We collected 492 *Acinetobacter* spp. strains from two hospitals in Taiwan and classified them using MALDI-TOF MS and *bla*_OXA-51-like_ PCR; 94.5% were *A. baumannii,* and 5.5% were non-*A*. *baumannii* (NAB). We confirmed their identity by *rpo*B gene sequencing of 239 randomly selected *A. baumannii* strains and all 27 NAB strains. Our analysis revealed that the *rpo*B alleles of OXA51-like-negative strains matched those of two NAB species, *A. seifertii* and *A. nosocomialis*, while all OXA51-like-positive strains matched *A. baumannii*, as per the Pasteur MLST scheme database. Among the 492 strains, 240 exhibited carbapenem resistance, including 237 carbapenem-resistant *A. baumannii* (CRAB) strains and three CR-NAB strains. All CRAB strains were positive for *bla*_OXA-51-like_; 72.6% also carried *bla*_OXA-23-like_, 22.8% carried *bla*_OXA-24-like_, 3.4% co-carried *bla*_OXA-23-like_+*bla*_OXA-24-like_, and 1.27% carried *bla*_OXA-51-like_ alone. Among the three CR-NAB strains, one carried *bla*_NDM-1_, and two co-carried *bla*_OXA-58-like_+*bla*_IMP_. We also established a new multiplex PCR method for rapid screening of common capsular types (KL), which showed a difference between CRAB and carbapenem-susceptible *A. baumannii* (CSAB). KL2, KL10, KL22, and KL52 accounted for 76.6% of CRAB strains, whereas about half of the CSAB strains were other KL types. Of the remaining CSAB strains, KL14 was the most predominant type at 10.3%. We further conducted MLST Pasteur typing for 262 isolates and found that the carbapenemase genes were correlated with either ST or KL types. Additionally, KL types showed correlations with ST types, carbapenem resistance, and certain clinical records. Whole-genome sequencing of a *bla*_NDM-1_-carrying *A. seifertii* strain revealed a plasmid transferable via *in vitro* conjugation, suggesting *A. seifertii* may be a reservoir for NDM-1 plasmids.

**IMPORTANCE:**

Carbapenem-resistant *Acinetobacter* spp. have been identified by the World Health Organization as a top priority for new antibiotic development. We established a rapid KL-typing method for efficient screening of *Acinetobacter baumannii* strains to enable epidemiological surveillance and provide a foundation for effective infection control. Our investigation of the molecular epidemiology of the *A. baumannii* group isolates revealed the prevalence of carbapenemase genes and major KL types among CR and CS strains of *A. baumannii* and NAB. We identified an *A. seifertii* strain carrying a Ti-type conjugative operon on a small plasmid that harbored genes encoding the NDM-1 carbapenemase alongside genes conferring resistance to aminoglycosides and bleomycin and closely resembled sequences detected in *A. soli* and *A. pittii* in Taiwan and China, respectively, suggesting its potential for transmitting multidrug resistance and contributing to the spread of antimicrobial resistance.

## INTRODUCTION

The *Acinetobacter baumannii* (AB) group, currently composed of *A. baumannii*, *A. pittii*, *A. nosocomialis*, *A. dijkshoorniae*, and *A. seifertii* (formerly *Acinetobacter* genomic species Close to 13TU), has emerged globally as prominent nosocomial pathogens (1–3). Infections with antibiotic-resistant *Acinetobacter* spp., especially carbapenem-resistant *A. baumannii* (CRAB) or multidrug-resistant *A. baumannii* (MDRAB), carry a higher risk of mortality than infections with susceptible strains, and their prevalence in nosocomial infections has been steadily rising (4). Resistance to carbapenems—last-resort therapeutics for MDRAB infections—is increasing, with resistance rates >80% in several countries (5). CRAB has been categorized as a critical priority pathogen by the World Health Organization for combatting antibiotic resistance and developing advanced therapies (6). Mechanisms of carbapenem resistance can be classified into two types: enzymatic inactivation and non-enzymatic (7). Non-enzymatic mechanisms involve modifying penicillin-binding proteins, losing outer membrane proteins, and forming biofilms. Enzymatic mechanisms are categorized into three classes (A, B, and D β-lactamases) based on their hydrolytic mechanism. In CRAB, the major mechanism is the acquisition and production of Ambler class D enzymes, which are also called oxacillinase enzymes (OXAs) (8). Common class D β-lactamase genes include the intrinsic *bla*_OXA-51-like_ genes and the acquired *bla*_OXA-23-like_, *bla*_OXA-24-like_, *bla*_OXA-58-like_, *bla*_OXA-134-like_, and *bla*_OXA-143-like_ genes, which are usually associated with mobile genetic elements (9). Metallo-β-lactamase (MBL) class B enzymes have been detected in *A. baumannii*, including first New Delhi metallo-β-lactamase (NDM-1) from India (10), imipenemase (IMP-1) from Brazil (11), Verona integron-encoded metallo-β-lactamase (VIM-1) from Greece (12), and Seoul imipenemase (SIM-1) from Seoul, Korea (13). The least common β-lactamase class in *A. baumannii* is the Ambler class A enzymes, including *Klebsiella pneumoniae* carbapenemase (KPC) and Guiana extended-spectrum (GES), which was first reported in Puerto Rico (14). Although OXA-type enzymes are more common in *A. baumannii* than MBLs, MBLs have 100–1000 times stronger carbapenem-resistance activities than OXA-type enzymes (15). Among carbapenem-resistant *Acinetobacter* spp. (CRA), IMP- and NDM-type enzymes are the most epidemiologically important and clinically relevant acquired MBLs (16). Globally, IMP-type enzymes have been detected in Enterobacteriaceae and Gram-negative non-fermenters (mostly *Acinetobacter* spp. and *P. aeruginosa*) (11). NDM-1, a recently identified acquired MBL, raises concerns due to its potential for global spread and has been reported in *Acinetobacter* spp., either plasmid- or chromosome-encoded, often alongside other resistance genes (17–20).

Capsule is another virulence determinant for CRAB (21). More than 200 capsular types (KL types) have been detected in *A. baumannii*, and the clinical significance of several, including KL1, KL2, KL3, KL6, KL9, KL10, KL14, KL22, KL47, KL49, and KL52, has been reported (22–28). Several reports have shown that capsular type is the predominant virulence factor (22, 24, 28). Based on our previous study of CRAB in Taiwan, four KL types (KL2, KL10, KL22, and KL52) are the most common and clinically important. Two other types, KL14 and KL-other, are common in carbapenem-susceptible *A. baumannii* (CSAB) (22). Morbidity and mortality are higher for patients with CRAB infections than for patients with CSAB infections. In addition, infection with the four major KL types (KL2/10/22/52) of CRAB resulted in significantly more severe outcomes than infection with other KL types. In this study, we developed a novel multiplex polymerase chain reaction (PCR) assay based on *wzy* genotyping to detect 12 KL types in *A. baumannii* that have been reported as predominant and/or clinically significant (KL1, KL2, KL3, KL6, KL9, KL10, KL14, KL22, KL47, KL49, KL52, and KL81). This rapid KL-typing method facilitates efficient screening of numerous *A. baumannii* strains, which will aid in epidemiological monitoring and establish a foundation for infection control. We investigated the prevalence of carbapenemase genes and KL types using this novel multiplex PCR method among clinical AB-group strains isolated from 2015 to 2021 in Taiwan. We also describe the first case of an infection caused by an NDM-1-producing *A. seifertii* strain in Taiwan and identify a plasmid harboring the *bla*_NDM-1_ gene in this strain.

## RESULTS

### Characteristics and classification of AB group isolates

**Fig 1:**
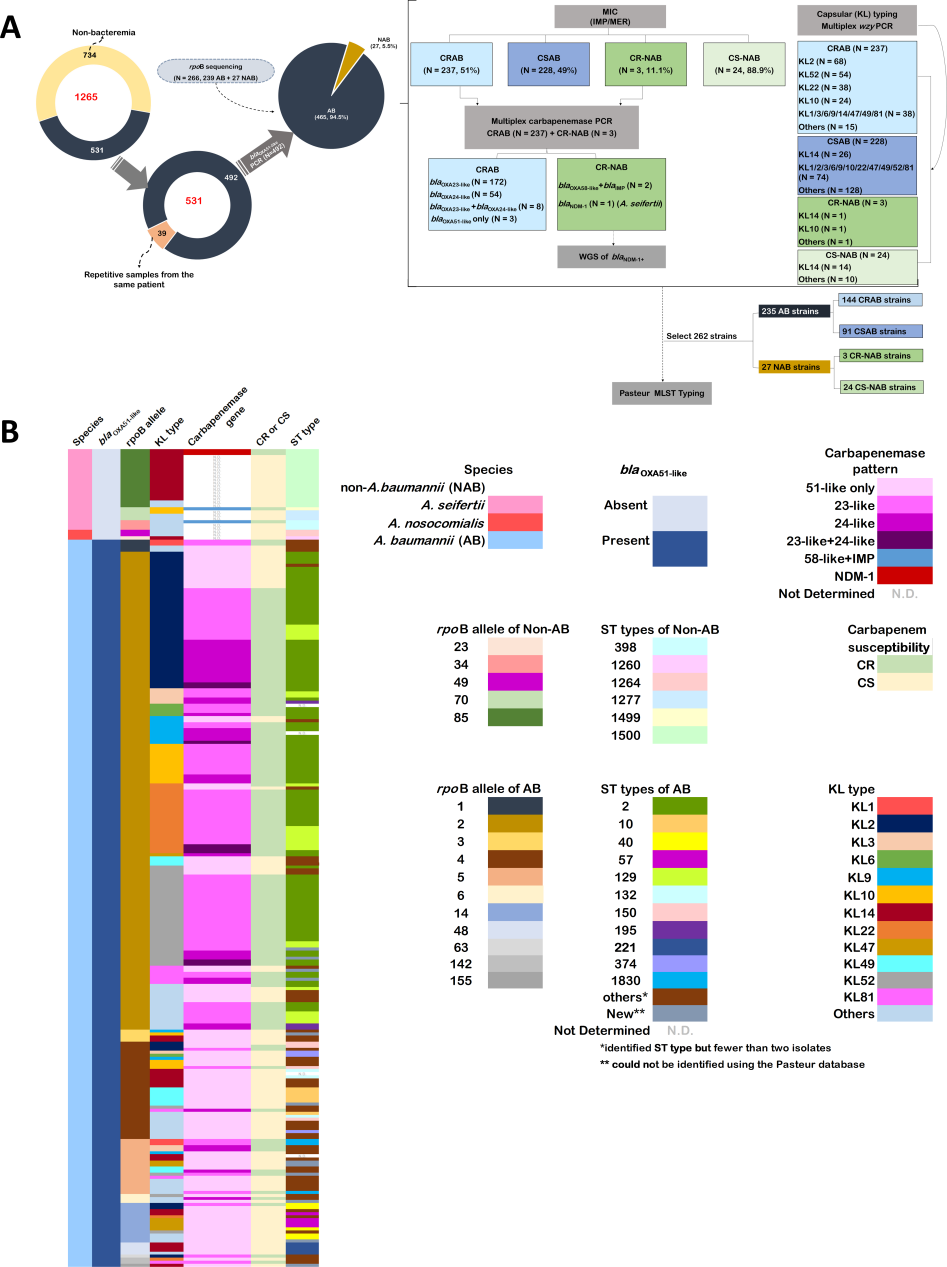
Genotyping characteristics of *Acinetobacter baumannii* (AB) group isolates. (**A**) Flowchart for the sample collection, genotypes, demographics, and species characteristics. A total of 492 samples from patients with bacteremia (either nosocomial or community-acquired) were analyzed in this study. Abbreviation: AB, *Acinetobacter baumannii*; NAB, non-*A*. *baumannii*; CRAB, carbapenem-resistant *A. baumannii*; CSAB, carbapenem-susceptible *A. baumannii*; CR-NAB carbapenem-resistant non-*A*. *baumannii*; CS-NAB, carbapenem-susceptible non-*A*. *baumannii*; MIC, minimum inhibitor concentration; IMP, Imipenem; MER, Meropenem; WGS, whole-genome sequence; MLST, multi-locus sequence type. (**B**) Capsular types (KL types), sequence types (ST types), and carbapenemase genes of 262 isolates, including 235 AB and 27 NAB isolates. The *rpo*B allele analysis (*n* = 266, including 239 AB + 27 NAB) was used to confirm species differentiation within the AB group.

Out of 1,265 AB-group clinical strains collected from 2015 to 2021, 734 non-bacteremia samples and 39 repetitive samples from the same patient were excluded, yielding a total of 492 non-redundant strains causing bacteremia (either nosocomial or community-acquired) that were included in this study (Fig. 1A; Table S1). The distribution of the 492 isolates across 12 months over a 7-year period is presented in Fig. S1. The number of infection cases did not show a notable increase in any particular month, suggesting that no evident outbreaks occurred during the study period. These strains were identified using MALDI-TOF MS (29, 30) and *bla*_OXA-51-like_ PCR (31, 32). The *bla*_OXA-51-like_ gene is considered highly specific to *A. baumannii*, with *A. baumannii* strains typically testing positive for this gene, while non-*A*. *baumannii* (NAB) strains are negative (31). Among 492 strains, 465 (94.5%) were *bla*_OXA-51-like_ positive (*A. baumannii*), and 27 (5.5%) were *bla*_OXA-51-like_ negative (NAB). Their characteristics are shown in Fig. 1A.

RNA polymerase β-subunit (*rpo*B) gene sequences are useful for identification and taxonomic classification within the AB group (2, 32, 33). Thus, we conducted *rpo*B sequencing to confirm their identity as *A. baumannii* or NAB. Sequencing of all 27 OXA-51-like-negative strains and 239 randomly selected OXA-51-like-positive strains showed that all 239 OXA-51-like-positive strains matched *A. baumannii*, whereas the OXA-51-like-negative strains matched *A. nosocomialis* (*n* = 3) and *A. seifertii* (*n* = 24) (NAB), which was consistent with the presence/absence of *bla*_OXA-51-like_. Among *A. baumannii* strains, rpoB-2 was the most prevalent variant (157/239), whereas rpoB-85 was the most common in NAB strains (17/27) (Fig. 1B).

### Carbapenem resistance determinants

**Fig 2:**
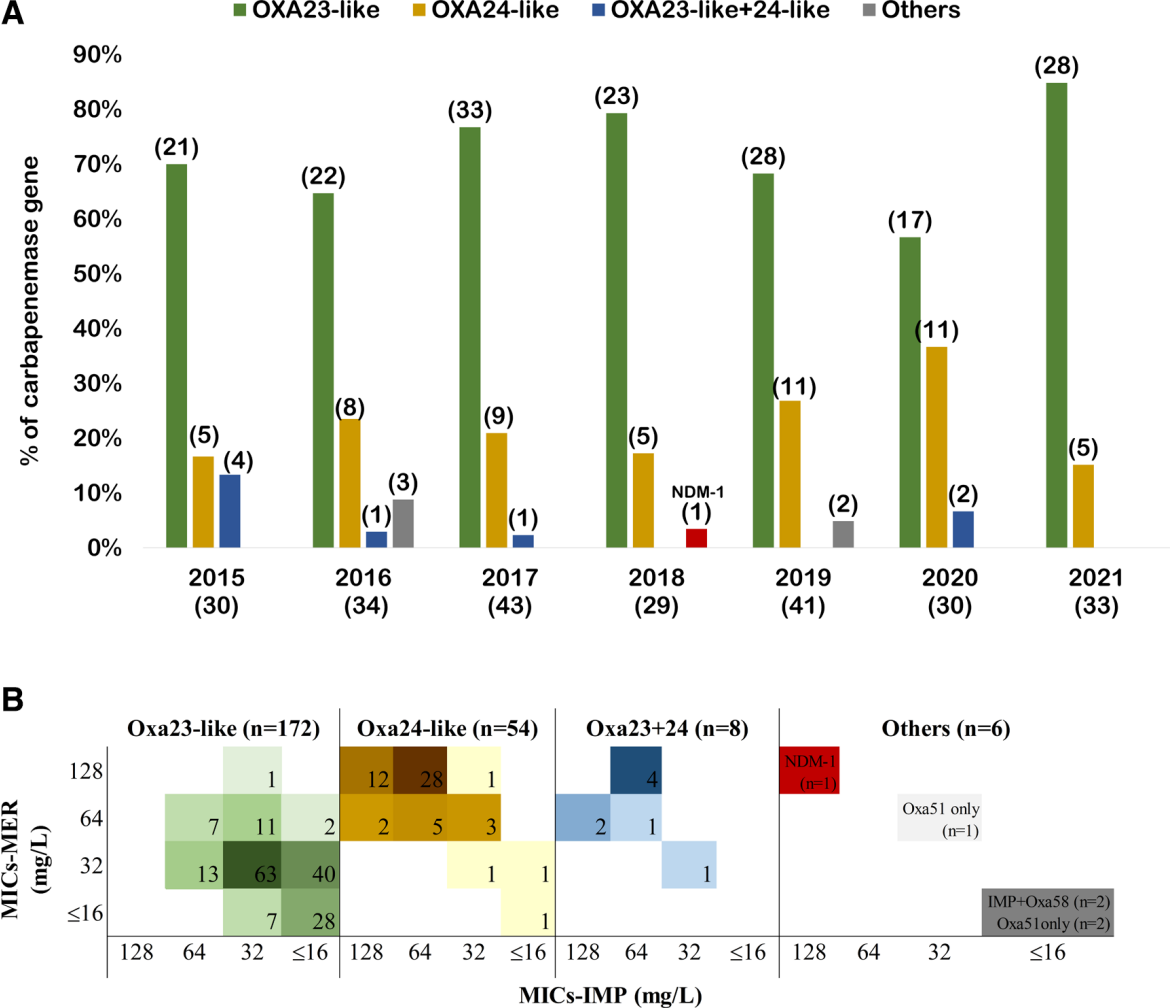
Carbapenemase-producing strains over 7 years in Taiwan. (**A**) The percentage of isolates carrying carbapenemase genes, *bla*_OXA-23-like_, *bla*_OXA-24-like_, *bla*_OXA-23-like_+*bla*_OXA-24-like_, *bla_N_*_DM-1_ (shown in red color), and others (*bla*_IMP+OXA-58_, *bla*_OXA-51-like_ only) in different years are displayed. The number of isolates is shown in parentheses. (**B**) MIC distribution of carbapenems among carbapenem-resistant (CR) isolates with different carbapenemase gene patterns (IMP, Imipenem; MER, Meropenem). The number of isolates carrying carbapenemase genes in different carbapenem MICs is displayed in each square.

We identified 48.8% of the 492 isolates as carbapenem-resistant, with minimum inhibitory concentrations (MICs) of imipenem (IMP) and meropenem (MER) >4 mg/L, including 237 CRAB strains and three CR-NAB strains. These strains were screened for carbapenemase genes, and *bla*_OXA-143-like_, *bla*_KPC_, *bla*_GES_, *bla*_VIM_, *bla*_SPM_, *bla*_GIM_, and *bla*_SIM_ were not detected. Among the 237 CRAB strains, 72.6% carried *bla*_OXA-23-like_, 22.8% carried *bla*_OXA-24-like_, 3.3% co-carried *bla*_OXA-23-like_ and *bla*_OXA-24-like_, and 1.3% harbored *bla*_OXA-51-like_ only (Fig. 1A). Among the three CR-NAB strains, two co-carried *bla*_OXA58-like_ and *bla*_IMP_, and one carried *bla*_NDM-1_ (Fig. 1A). Oxa-23-like and Oxa-24-like were the two major carbapenemases, and no apparent difference was observed between 2015 and 2021 (Fig. 2A). Notably, NDM-1 first appeared in 2018.

The MICs of IMP and MER were compared in four groups, each harboring different carbapenemase genes: *bla*_OXA-23-like_, *bla*_OXA-24-like_, *bla*_OXA-23-like_+*bla*_OXA-24-like_, and others. We observed that 87% (47/54) of *bla*_OXA-24-like_-carrying strains and 87.5% (7/8) of *bla*_OXA-23-like_+*bla*_OXA-24-like_ co-carrying strains had IMP and MER MICs of 64 mg/L or greater; in contrast, only 4.1% (7/172) of *bla*_OXA-23-like_-carrying isolates had IMP and MER MICs of 64 mg/L. These results showed that *bla*_OXA-24-like_-carrying and *bla*_OXA-23-like_+*bla*_OXA-24-like_ co-carrying strains had higher IMP and MER MICs than *bla*_OXA-23-like_-carrying strains (Fig. 2B). Notably, the strain carrying *bla*_NDM-1_ had high MICs of both IMP and MER (128 mg/L), whereas the strain carrying *bla*_OXA-58-like_+*bla*_IMP_ showed a relatively low MIC (≤16 mg/L) (Fig. 2B).

### Multiplex *wzy* PCR for rapid capsular typing

Variations in KL types have been reported in different geographic areas; however, some types are predominant in clinical collections or MDR/CRAB worldwide. A literature search showed a high prevalence of certain KL types of *A. baumannii*, including KL1 in Afghanistan (34); KL2 and KL9 in the USA (26); KL2, KL10, KL14, KL22, and KL52 in Taiwan (22); KL3 and KL49 in China (27); and KL2, KL49, and KL58 in Vietnam (25). In contrast, KL6, KL10, and KL47 were common types of global clone (GC) 2 in Thailand (23). Recently, Cahill et al. reported 237 KL-type reference sequences, including 145 new KL types in the *Kaptive* database, and that KL2 was the most common type, followed by KL3 and KL22 (35). Four major KL types (KL2/10/22/52) of CRAB were associated with increased disease severity and mortality in Taiwan (22).

**Fig 3:**
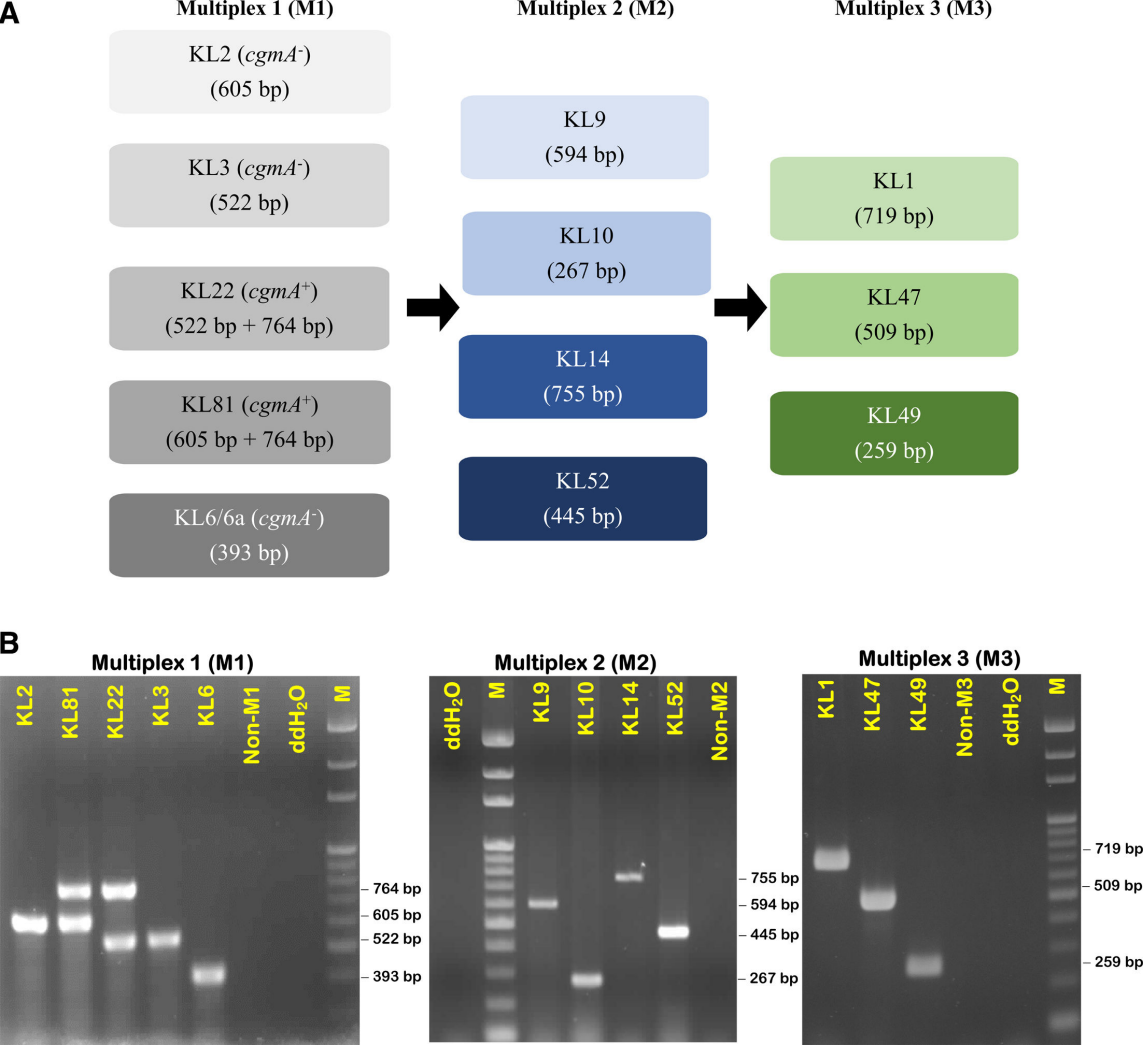
Multiplex *wzy* PCR for rapid capsular typing. (**A**) Flow diagram of capsular typing using multiplex-three-stages *wzy* PCR. The size of each amplicon is indicated in text boxes. (**B**) Agarose gel electrophoresis (1.7%) was used for the separation of the different multiplex *wzy* PCR products. M1 multiplex detecting KL2 (KL2/81^+^ + *cgm*A^-^), KL81 (KL2/81^+^ + *cgm*A^+^); KL3 (KL22/3^+^ + *cgm*A^-^), KL22 (KL22/3^+^ + *cgm*A^+^); KL6 (KL6^+^ + *cgm*A^-^). M2 multiplex detecting KL9; KL10; KL14; KL52. M3 multiplex detecting KL1; KL47; KL49; lane “non−M1/2/3”, the PCR reaction was a mixture of M1, M2, or M3 primers with DNA of KL33. ddH_2_O was used as a negative control. M, 100 bp DNA ladder.

Here, we selected 11 common KL types with potential clinical significance either in Taiwan or in more than one other country (KL1, KL2, KL3, KL6, KL9, KL10, KL14, KL22, KL47, KL49, and KL52) and developed a multiplex *wzy* PCR to rapidly detect them. The method can ultimately detect 12 types because KL2 can be differentiated from KL81. The primers used are listed in Table 1 and were tested in a multiplex approach, which was divided into three stages (Fig. 3A). Twelve clinical isolates, one representing each of the 12 types, were used as positive controls to evaluate the expected bands and confirm the utility of the PCR primers. In the M1 multiplex assay, four primer pairs were used: KL2/KL81 *wzy*, KL3/KL22 *wzy*, KL6 *wzy*, and *cgm*A. The *cgm*A gene was used to distinguish KL2 from KL81 and KL3 from KL22 because the only difference between both KL2/KL81 and KL3/KL22 is the presence of *cgm*A, i.e., when a strain is KL2/KL81 *wzy* positive, it can be distinguished as KL81 if it is *cgm*A+ or KL2 if it is *cgm*A−. Similarly, when a strain is KL3/KL22 *wzy* positive, it can be distinguished as KL22 if it is *cgm*A+ or KL3 if it is *cgm*A−. Strains that were negative for M1 were subjected to the next PCR, M2, and so on. The results showed no expected products for type KL33 (negative control) and positive reactions only for the corresponding KL types, indicating that our assay is useful and reliable for the identification of the 12 aforementioned KL types (Fig. 3B).

**TABLE 1:** Oligonucleotides of *wzy* multiplex PCR used in this study

Stage	Primer name	Sequence 5′−3′	Product size (bp)	Purpose	Reference
(M1)	KL22/3_F	GGAGTAGAGATTGGTTGGG	522	KL22 and KL3 detection	^*a*^
	KL22/3_R	GCCAACACTTTCAGCATAATC			
	KL2/81_F	TTTTCTCCTGTTTGATGGGG	605	KL2 and KL81 detection	^*a*^
	KL2/81_R	AAATCAGCATTCCAGCGCAC			
	KL6_F	GGTTCTTAAGGTTGCTTGCTCT	393	KL6 detection	This study
	KL6_R	ACACCTTTGCAGTATGGCG			
	*cgm*A-F	CTTTTGGTGGAGGTACGGCG	764	Differentiate KL2 and KL81 or KL22 and KL3	This study
	*cgm*A-R	AGGCAACCCTGCTAGCTTTA			
(M2)	KL9_F	TACTTTGGGCTTGTGTGGG	594	KL9 detection	^*a*^
	KL9_R	AGCCACTCGTTCTACATCC			
	KL10_F	AGGACGACTATTATTGGTATTGTGT	267	KL10 detection	This study
	KL10_R	AGCATCACCTAAAATCCAAGTTCT			
	KL14_F	ATGAATTGTATTGGGGGCAG	755	KL14 detection	This study
	KL14_R	TTGGCTTCCTGTCACTAAAC			
	KL52_F	ACAGGGTTTTGCTGTTGCAG	445	KL52 detection	^*a*^
	KL52_R	ACCTAAGCGAAAACCTAACC			
(M3)	KL1_F	TTCTTTAGGGGTGTTTGGTG	719	KL1 detection	^*a*^
	KL1_R	GTAACACCTCCCCCATCATC			
	KL47_F	TGGCTATCTGGCTTTGCAC	509	KL47 detection	^*a*^
	KL47_R	GTGAAACGGCACCCAAAAG			
	KL49_F	TTTTTGACTAGGCAAGGGGC	259	KL49 detection	This study
	KL49_R	AAAAGATTGTGGGCAAGCC			

^*a*^ Previous study (22).

### Distribution of KL types from 2015 to 2021

**Fig 4:**
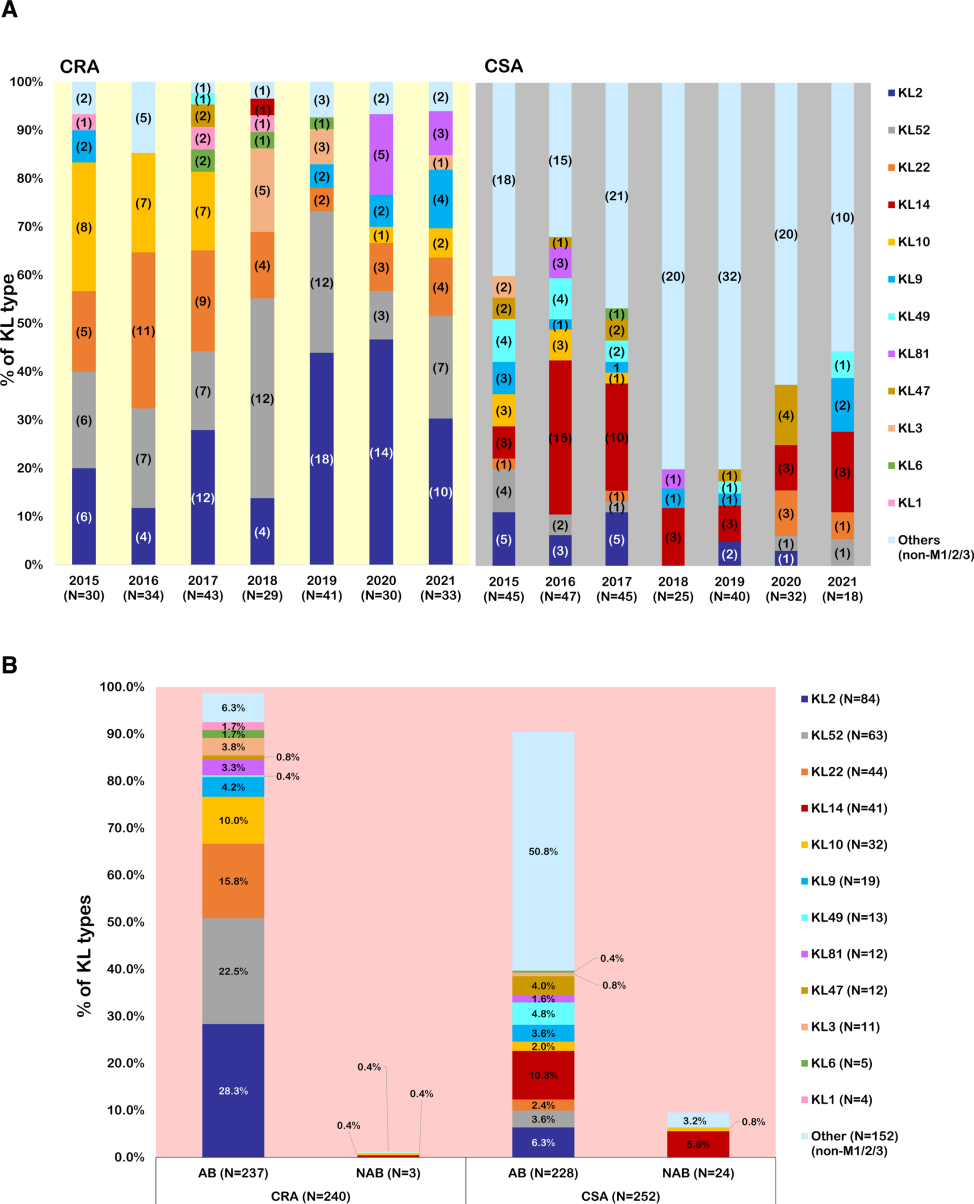
Percentage of variants of capsular types (KL) among 492 *Acinetobacter* spp. isolates. (**A**) The distribution KL of carbapenem-resistant *Acinetobacter* spp. (CRA) and carbapenem-susceptible *Acinetobacter* spp. (CSA) from 2015 to 2021. The number of isolates is shown in parentheses. (**B**) The comparison of variants of KL types between *A. baumannii* (AB) and non-*A*. *baumannii* (NAB) among carbapenem-resistant *Acinetobacter* (CRA) and carbapenem-susceptible *Acinetobacter* (CSA) isolates.

The KL typing results of the 492 isolates revealed no obvious trends (Fig. 4A). However, there were variations in some types. For example, KL10 was predominant in 2015–2017 but was absent in 2018–2019 and reappeared in 2020–2021. Similarly, KL9 was present in 2015 and 2019–2021 but was absent in 2016–2018; KL6 was present in 2017–2019 but was a minor type. Despite KL1 being reported as the prevalent type in GC1 (not commonly found in Asia), we found four KL1 strains among the CRA isolates appearing in 2015, 2017, and 2018. Interestingly, KL3 emerged during 2018–2019, and KL81 emerged during 2020–2021. Finally, KL2, KL22, and KL52 were consistently prevalent throughout the 7-year period.

Among the 240 CRA isolates, four types (KL2/10/22/52) collectively accounted for 76.6% of the total isolates; KL2 was the most prevalent at 28.3%, followed by KL52 at 22.5%, KL22 at 15.8%, and KL10 at 10%. Less frequently encountered isolates were KL9 (4.2%), KL3 (3.8%), and KL81 (3.3%) (Fig. 4B). About half of the CSA isolates were other KL types; among the remaining types, KL14 was the most predominant, representing 10.3% of CSAB and 5.6% of CS-NAB isolates. Because half of the CSA isolates were other KL types, we cannot exclude the possibility that an unidentified KL type accounts for the majority of CSA isolates.

### Correlation among KL types, ST types, and carbapenemase genes

To explore the correlation between KL types, ST types, and carbapenemase gene patterns, we selected a representative subset of 262 of 492 strains, including 235 *A*. *baumannii* isolates (144 CRAB and 91 CSAB) and all NAB isolates for Pasteur MLST typing. The criteria for selecting bacterial strains for this experiment are detailed in the Supplemental Methods. The ST types for the 262 strains are shown in Fig. 1B; Table S1. Among the 235 *A*. *baumannii* strains, ST2 was the most prevalent at 47.7% (*n* = 112), followed by ST129 at 10% (*n* = 23), while less common types included ST10 (*n* = 6), ST40 (*n* = 5), ST57/ST150/ST221 (*n* = 4 each), ST132/ST195/ST374/ST1830 (*n* = 3 each), along with 53 other types with no more than two strains each and 12 new types that were unidentified using the Pasteur database. For 27 NAB strains, ST1500 was most prevalent (*n* = 17), followed by ST1277 and ST398 (three each), ST1264 (2), and ST1499/ST1260 (one each).

The carbapenemase gene patterns showed a correlation with either KL or ST types. For example, all KL1 strains exhibited *bla*_OXA-23-like_ patterns (Table S2). Moreover, *bla*_OXA-23-like_ accounts for the majority pattern for several KL types, particularly dominating over other carbapenemase patterns in KL2, KL10, KL22, and KL52. Interestingly, *bla*_OXA-24-like_ was primarily identified in KL2. Similarly, the *bla*_OXA-23-like_ pattern was predominant in many ST types except for ST195, which all possessed *bla*_OXA-24-like_. Notably, none of ST129 (a major GC) exhibited *bla*_OXA-24-like_ pattern (Table S3). To further assess ST clustering of CRAB strains, we used the web-based tool PHYLOViZ (https://www.phyloviz.net/) to analyze the genetic relationships among 144 CRAB strains with identified ST types. Our analysis revealed that strains carrying additional *bla*_OXA_ genes (*bla*_OXA-23-like_ or *bla*_OXA-24-like_ or both) primarily clustered into two major groups, ST2 and ST129, though some strains were dispersed among other ST types (Fig. S2A). Additionally, higher MIC values were not linked to specific ST types; instead, they were associated with *bla*_OXA-24-like_ carriage, as we previously noted. The association between KL and ST types was also noted (Table S4). ST2 was predominant across multiple KL types, including KL2, KL9, KL10, KL22, and KL52, while ST10, ST57, and ST1500 were predominant in KL49, KL47, and KL14, respectively. Additionally, the ST clustering of various KL types is visualized in Fig. S2B, which illustrates that some KL types had strains that clustered closely, while others were more diverse. For instance, KL9 (*n* = 11) predominantly clustered with ST2 (*n* = 7), with the remaining four strains scattered across different ST types. In contrast, KL1 was evenly associated with two ST types, ST20 (*n* = 2) and ST1830 (*n* = 2). To further understand the relationships between strains with the same KL types, detailed evaluations using WGS analysis are necessary.

### The association between capsular types (KL types) and general clinical factors

The association between KL types and age, gender, hospitals, or carbapenem resistance was evaluated. The results revealed that the distribution of KL types differed based on carbapenem susceptibility and gender (Table S5). We also found that most M1/2/3 KL types were associated with carbapenem resistance, except for KL14, KL47, and KL49, which were more likely to be carbapenem-susceptible. Notably, there was no significant difference in the distribution of KL types between the two hospitals (Table S5).

### Nosocomial infections (NIs) and community-acquired infections (CAIs)

The difference between nosocomial infections (NIs) versus community-acquired infections (CAIs) was further analyzed across various factors. The results revealed that the distribution of KL types varied between NI and CAI. Notably, KL1 was exclusively detected in NI, while KL10, KL14, KL22, KL47, and KL52 were more frequently observed in NI than in CAI. In contrast, KL6 and KL49 were more commonly found in CAI than in NI. Besides, more strains belong to non-M1/M2/M3 types in CAI (44%) compared with those in NI (28.8%) (Table S6). Similarly, the distribution of ST types differed between NI and CAI. Some ST types were unique to NI. The top three ST types for NI were ST2 (45%), ST129 (9%), and ST1500 (7.7%), with ST2 being particularly dominant. In contrast, for CAI, the most common ST types were ST2 (27.5%), ST10 (12.5%), and ST129 (7.5%) (Table S7). Additionally, a correlation was found between carbapenem resistance and infection types, with higher resistance rates in NI compared with CAI, while CAI patients tended to be older than those with NI (Table S8).

### WGS analyses and antimicrobial resistance genes of NDM-1-carrying *A. seifertii* isolates

Among our collection, an *A. seifertii* strain positive for NDM-1 (AS39) has high MICs (128 mg/L) of IMP and MER. Because NDM-1 is rarely found in *Acinetobacter* spp. in Taiwan, we further investigated the genetic background of the strain using WGS (accession no. CP134858- CP134863). In accordance with the results of the multiplex *wzy* PCR approach, AS39 was identified as KL14, and comparison to the *Kaptive* database showed high similarity to the KL14 *cps* locus, with 93% identity and 100% coverage. The AS39 genome is comprised of a single 4.05 Mb chromosome and five plasmids designed pAS39-1 (~135.9 kb), pAS39-2 (~47.2 kb), pAS39-3 (~11 kb), pAS39-4 (~11 kb), and pAS39-5 (~8 kb) (Table 2). Typing showed that AS39 is ST1500 and ST-new under the Pasteur and Oxford schemes, respectively. AS39 is closely related to *A. seifertii* isolate TUM15118 (accession no. BKHW00000000) from Japan, with >95% identity and >90% coverage (TUM15118 is No. 6053 shown in Fig. S3). Remarkably, *A. seifertii* No. 6053 lacks the presence of carbapenemase genes, whereas AS39 is a CRA strain harboring the NDM-1 plasmid, indicating that *A. seifertii* may have spread from country to country.

**TABLE 2:** Genome features of AS39*^d^*^,^*^e^*

ST-type	KL type / OCL type	Replicons	Size (bp)	Plasmid name	Carbapenemase gene(s)	Other resistance gene(s)/ efflux-mediated antimicrobial resistance genes	Accession no.
1500^Pas^ / New^Oxf^	KL14 / OCL12	Chromosome	4,053,050	-	-	*bla*_ADC-5_, *eptA*, *parY* RND^*a*^ family: (*adeA, adeB, adeR, adeS), (acrB, acrD),* (*adeI, adeJ, adeK, adeN), (adeL, adeF, adeH), ceoA, acrD, mdtC, MuxB, smeR, mexK* MFS^*b*^: *emrA, (rosA, rosB*) Others^*c*^: *abeS* (SMR), *msbA* (ABC), *macB* (ABC), abeM (MATE)	CP134858
		Plasmid	134,257	pAS39-1	-		CP134859
		Plasmid	47,271	pAS39-2	*bla*_NDM-1_	*aph(3’*)-VI, *ble*_MBL_	CP134860
		Plasmid	10,753	pAS39-3	-		CP134861
		Plasmid	10,261	pAS39-4	-		CP134862
		Plasmid	7,137	pAS39-5	-		CP134863

^*a*^ RND, resistance–nodulation–cell division antibiotic efflux pump.

^*b*^ MFS, major facilitator superfamily antibiotic efflux pump.

^*c*^ SMR, small multidrug resistance antibiotic efflux pump; ABC, ATP-binding cassette antibiotic efflux pump; MATE, multidrug and toxic compound extrusion transporter.

^*d*^ Genes in parentheses and underlined indicate that they are part of an operon.

^*e*^ "-" means "Undetermined".

Antimicrobial resistance genes were identified using the Comprehensive Antibiotic Resistance Database. Most acquired antimicrobial resistance genes in AS39 were located on the chromosome (Table 2). AS39 was resistant to carbapenems (imipenem and meropenem), cephalosporins (cefazolin, ceftazidime, ceftriaxone, and cefepime), aminoglycosides (gentamicin and amikacin), and was susceptible to colistin and tigecycline. The *adc*-5 gene, encoding extended-spectrum AmpC cephalosporinase, was identified as the gene conferring cephalosporin resistance, consistent with the antibiotic susceptibility results, which showed that AS39 is resistant to cephalosporins (Table S9). Increased cephalosporin resistance has been linked with the upregulation of *bla*_ADC_ genes through the insertion of IS*Aba*1 or related IS elements that harbor outward-directed promoters (36); however, no IS elements were found upstream of the *bla*_ADC-5_ gene in AS39. The AS39 chromosome also harbors *ade*RS and *ade*AB but lacks *ade*C; these three genes are believed to encode an efflux pump in the multidrug resistance–nodulation–cell division (RND) family efflux system along with a two-component regulatory system, *ade*RS, which is located upstream and is transcribed divergently from *ade*ABC. This efflux system is usually chromosomally encoded in *Acinetobacter* strains, and overexpression of *ade*ABC was correlated with multidrug resistance (37–39). The AS39 chromosome also harbors genes encoding other *Acinetobacter* RND family efflux pumps, including *ade*FGH and *ade*IJK, along with their respective regulatory genes *ade*L and *ade*N. Additionally, AS39 also has three genes (*ros*A, *emr*A, and *ros*B) encoding efflux pumps of the major facilitator superfamily (MFS) and *abe*S, which encodes a small-multidrug resistance (SMR) family efflux pump. These antibiotic efflux pumps confer resistance to multiple drug classes, including fluoroquinolones, aminoglycosides, cephalosporins, carbapenem, and tetracyclines (38, 40). In addition, genes encoding the lipid A phosphoethanolamine transferase *ept*A, the aminocoumarin self-resistant gene *par*Y, *abe*M-encoding multidrug and toxic compound extrusion (MATE) transporter, and ATP-binding cassette (ABC) antibiotic efflux pump-encoding genes, such as *mac*B and *msb*A, were also identified in the AS39 chromosome. Notably, AS39 carries the lipid A modification gene *ept*A, which is associated with colistin resistance due to its overexpression caused by the insertion of an IS*Aba1* element upstream of the gene (41–43). However, the strain was susceptible to colistin, which could be attributed to the lack of an upstream IS*Aba1* element.

### Characteristics of plasmid pAS39-2

**Fig 5:**
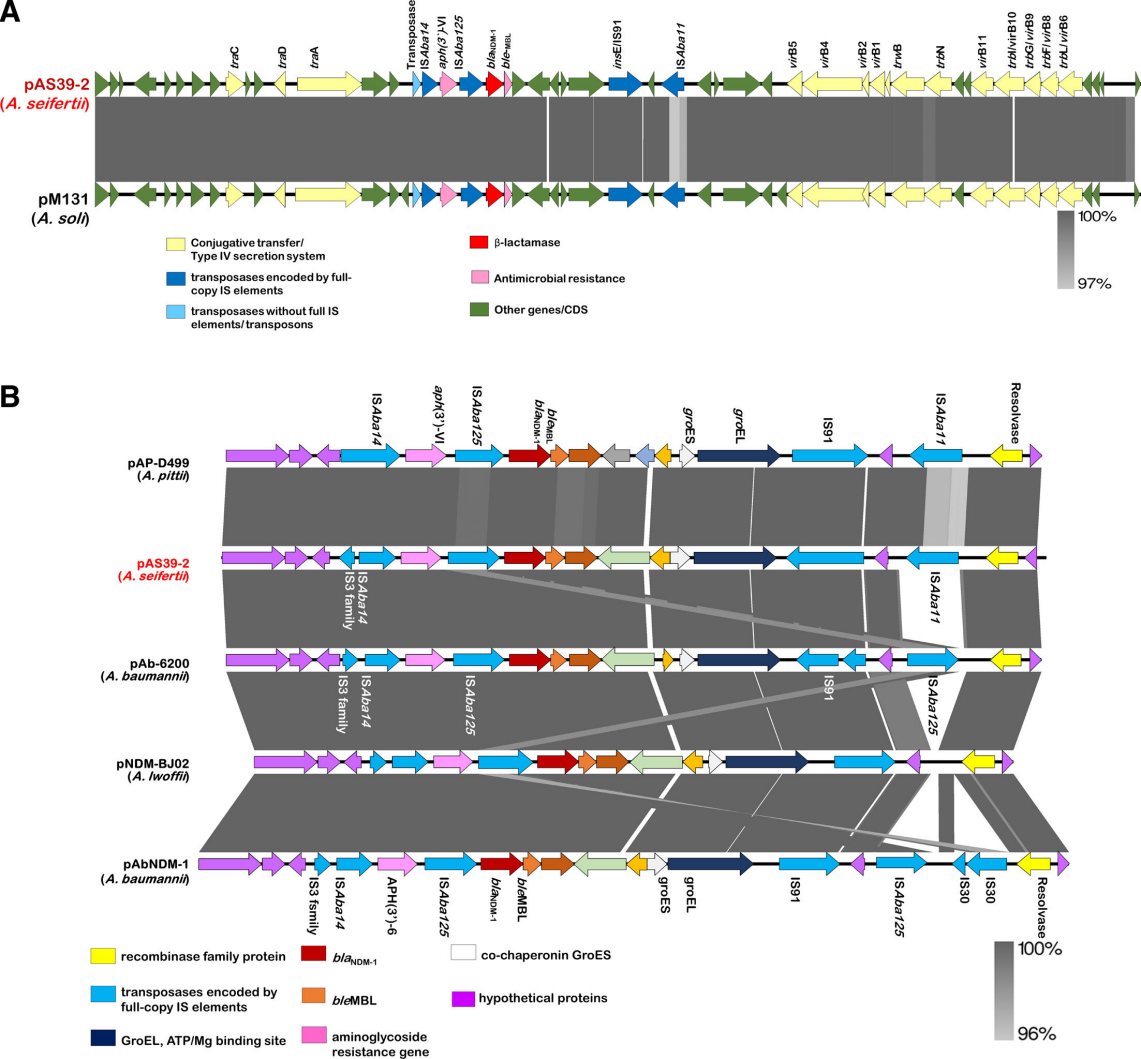
Comparison of pAS39-2 and other NDM-1-positive plasmids. (**A**) Comparative linear map of plasmid pAS39-2 and pM131_NDM1 plasmids. Arrows indicate the extent and directions of genes and ORFs. The *bla*_NDM-1_ gene is colored in red, and the other genes are colored according to their annotated functions: pink, antimicrobial resistance genes; yellow, genes with homology to conjugative transfer or type IV secretion system genes; dark blue and light blue, transposases encoded by full-copy IS elements and transposases without their corresponding IS elements or transposons in full, respectively; green, other functions and hypothetical protein-encoding genes. The extent of regions with >99% nucleotide sequence identities is indicated in the gray-shaded area. (**B**) Comparative map of the regions surrounding the *bla*_NDM-1_ gene in other *Acinetobacter* plasmids. Arrows indicate the extent and directions of the genes and ORFs. Accession numbers for the comparative map are as follows: pAP_D499 (accession no. NZ_AGFH01000030; nt 7,154–28,210); p6200-47.274kb (accession no. CP010399; nt 37,973–47 and continued from nt 3–10,798); pNDM-BJ02 (accession no. JQ060896; nt 454–20,404); pAbNDM-1 (accession no. JN377410; nt 454–22,697). The extent of regions with nucleotide sequence identities of between 96% and 100% is shown in gray.

Genome analysis showed that plasmid pAS39-2 has 42.3% GC content and contains 47 ORFs. Plasmid(s) in GenBank with the highest nucleotide similarities are shown in Table 3. Plasmid pAS39-2 is almost identical to pM131 (JX072963), which was isolated from *A. soli* in Taiwan (44), and pAP_D499 (NZ_AGFH01000030), which was isolated from *A. pittii* in China (45) (Fig. 5A; Fig. S4). Plasmid pAS39-2 contains *aph*(3′)-VI and *ble*_MBL_ genes, which provide resistance to aminoglycoside antibiotics (such as gentamicin, amikacin, and tobramycin) and bleomycin, respectively. The genetic content in the *bla*_NDM-1_ region of pAS39-2 was almost identical to that described previously (45), including genes encoding the GroES and GroEL chaperonin proteins, the gene encoding an IS91 family transposase, a gene encoding the protein-disulfide reductase DsbD family protein, and the gene encoding phosphoribosyl anthranilate isomerase, which is located downstream of *bla*_NDM-1_ (Fig. 5B). Additionally, the *bla*_NDM-1_ region of pAS39-2 was broadened from IS*Aba125* to IS*Aba11*, a discovery that deviated from previous reports in *A. baumannii*, in which it was typically flanked by two IS*Aba125* elements (17, 19, 46, 47) (Fig. 5B).

**TABLE 3:** Genbank plasmid(s) with the highest similarity

Query plasmid	Size (bp)	Matched plasmid	Accession no.	Species	Accession length (bp)	Identity	Coverage	Isolation source	Origin country
pAS39-1	134257	pAS73-1	CP061562	*Acinetobacter seifertii*	113,715	99.94%	81%	Blood	Taiwan
		pAS70-2	CP061573	*Acinetobacter seifertii*	114,524	99.73%	83%	Blood	Taiwan
		pAS74-2	CP061558	*Acinetobacter seifertii*	107,550	99.93%	81%	Blood	Taiwan
pAS39-2	47271	pM131_NDM1	JX072963	*Acinetobacter soli*	47,271	99.97%	100%	Sputum	Taiwan
		pAP_D499	NZ_AGFH01000030	*Acinetobacter pittii*	47,101	99.95%	100%	ICU surfaces	China
		p6200-47.274kb	CP010399	*Acinetobacter baumannii*	47,274	100%	97%	Bodily fluid	Columbia
		pAbNDM-1	JN377410	*Acinetobacter baumannii*	48,368	100%	97%	-	China
		pNDM-BJ01	JQ001791	*Acinetobacter lwoffii*	47,274	100%	97%	Urine	China
		pNDM1_010034	CP032278	*Acinetobacter sp*.	49,649	100%	97%	Sewage	China
pAS39-3	10753	p3VB280820	CP098794	*Acinetobacter baumannii*	10,753	99.98%	100%	Blood	India
		p4VB280821	CP098799	*Acinetobacter baumannii*	10,753	99.78%	100%	Blood	India
pAS39-4	10261	pAhaem2126chb	CP031996	*Acinetobacter haemolyticus*	10,261	100%	100%	Ascites liquid	Mexico
		p8_010062	CP033128	*Acinetobacter wuhouensis*	10,261	100%	100%	Sewage	China
		pBspH14	CP055282	*Acinetobacter* spp.	10,261	100%	100%	Soil	USA
		unnamed	CP077307	*Acinetobacter pittii*	10,261	100%	100%	Ear discharge	Germany
pAS39-5	7137	pAS73-3	CP061564	*Acinetobacter seifertii*	7,137	99.98%	100%	Blood	Taiwan
		pAS70-5	CP061576	*Acinetobacter seifertii*	7,137	99.89%	100%	Blood	Taiwan

### Transmissibility and stability of plasmid pAS39-2

Owing to its flexible and adaptable genome, *Acinetobacter* spp. are prone to accumulating antibiotic resistance determinants through horizontal gene transfer mechanisms, including transduction, conjugation, and natural transformation (17, 48–50). Therefore, we tested the conjugative ability of pAS39-2 using *A. seifertii* strain AS39 as a donor and 20 randomly selected *A. baumannii* strains as recipients. *K. pneumoniae* 17CRE24 (donor) and *E. coli* J53 (recipient), which are known to transfer a plasmid via conjugation, were used as a control (51). The results showed that AS39 could successfully transfer the *bla*_NDM-1_-carrying plasmid (pAS39-2) to *A. baumannii* strains, and the transconjugants (Ab (1–4)-*bla*_NDM-1_) exhibited higher MICs of IMP and MER (≥64 mg/L) than the recipient strains, which had a MIC of <1 mg/L (Table 4; Fig. S5). For the *A. baumannii* transconjugants, the transfer efficiency ranged from 1 × 10^−8^ to 3 × 10^−8^. In contrast, no transconjugant of *E. coli* J53 was obtained (Table 4). Furthermore, we attempted to transfer pAS39-2 from *A. baumannii* to *A. baumannii* using an *A. baumannii* transconjugant as the donor; however, no transconjugant was successfully obtained.

**TABLE 4:** Conjugal transfer of pAS39-2*^a^*^,^*^b^*

Strain	Bacteria species	Description	MICs (mg/L)		Resistance gene	Differentiate	
			IMP	MEM		*Wzy* PCR	*bla*_OXa51-like_ PCR
AS39	*A.seifertii*	Donor-KL14	128	128	*bla*_NDM-1_	KL14	-
Ab1	*A.baumannii*	Recipient-KL52	0.25	0.5	-	KL52	+
Ab1-*bla*_NDM-1_	*A.baumannii*	Transconjugant	128	128	*bla*_NDM-1_	KL52	+
Ab2	*A.baumannii*	Recipient-KL14	0.25	0.25	-	KL14	+
Ab2-*bla*_NDM-1_	*A.baumannii*	Transconjugant	128	128	*bla*_NDM-1_	KL14	+
Ab3	*A.baumannii*	Recipient-KL14	0.5	0.5	-	KL14	+
Ab3-*bla*_NDM-1_	*A.baumannii*	Transconjugant	64	128	*bla*_NDM-1_	KL14	+
Ab4	*A.baumannii*	Recipient-KL2	0.5	0.5	-	KL2	+
Ab4-*bla*_NDM-1_	*A.baumannii*	Transconjugant	64	128	*bla*_NDM-1_	KL2	+
17CRE24	*K. pneumoniae*	Donor	32	16	*bla*_OXA-48_		
J53	*E. coli*	Recipient	0.25	0.125	-		
J53-*bla*_OXA-48_	*E. coli*	Transconjugant	16	16	*bla*_OXA-48_		

^*a*^ Shown in gray as a control: *K. pneumoniae* 17CRE24 (donor) and *E. coli* J53 (recipient), which have been known to transfer a *bla*_OXA-48_ plasmid via conjugation, were used as the control for this assay.

^*b*^ "-" means absence; "+" means presence.

The stability of pAS39-2 in the donor and transconjugants was evaluated over 10 days of serial passage in the absence of carbapenem. Sudden loss of the *bla*_NDM-1_ plasmid was observed in the donor and transconjugants on day 1, and only a small percentage had maintained the plasmid at the end of the experiment, with plasmid-positive pAS39-2, Ab1-*bla*_NDM-1_, Ab2-*bla*_NDM-1_, Ab3-*bla*_NDM-1_, and Ab4-*bla*_NDM-1_ rates of 8%, 2.4%, 1.2%, 1.3%, and 1.7%, respectively (Fig. S6). This suggests that maintenance of the *bla*_NDM-1_ plasmid was markedly reduced in the absence of antibiotic-selective pressure, which echoes the notion that environmental changes, such as the absence of antibiotic-selective pressure or temperature, can impact the stability and maintenance of plasmids harboring antibiotic resistance genes (52–54).

## DISCUSSION

*A. baumannii* is more prevalent in patients who have adverse outcomes and increased resistance to antimicrobial agents than other pathogens within the AB group (50, 55); *A. pittii* and *A. nosocomialis* are clinically important and have been implicated in both community- and hospital-acquired infections. Recent studies have shown that *A. seifertii* and *A. dijkshoorniae* are novel, emerging pathogens capable of causing severe infections in humans (1, 56). Our results indicated that *A. baumannii* was more prevalent than NAB (Fig. 1A), similar to observations in other studies (57). *A. seifertii* was the major NAB species, suggesting its rise among clinical isolates in Taiwan.

Class D β-lactamase was the predominant class of carbapenemases in our CRAB clinical isolates, which is consistent with previous reports in Taiwan (58–61). Previous studies in Taiwan showed varying prevalences of *bla*_OXA-23-like_ and *bla*_OXA-24-like_ but with increasing trends in both. Lin et al. reported a prevalence of 4.2% for both *bla*_OXA-23-like_ and *bla*_OXA-24-like_ among 24 CRAB isolates collected in 2006 (57), and Chuang et al. reported 8% for *bla*_OXA-23-like_ and 12% for *bla*_OXA-24-like_ among 26 CRAB collected from the same year (62). Kuo et al. examined 72 CRAB strains isolated in 2007 and reported prevalences of 58% and 3% for *bla*_OXA-23-like_ and *bla*_OXA-24-like_ genes, respectively (59). Another study of 555 CRAB isolates collected from 2002 to 2010 in Taiwan found that isolates with *bla*_OXA-23-like_ and *bla*_OXA-24-like_ genes emerged in 2006, and their prevalences increased rapidly from 4.3% and 8.5% in 2006 to 78.8% and 15.9% in 2010, respectively (60). In our study, the prevalences of *bla*_OXA-23-like_ and *bla*_OXA-24-like_ in 2015 were approximately 70% and 17% (Fig. 2A), respectively, which are consistent with previous studies documenting a significant increase in *bla*_OXA-23-like_ after 2006 in Taiwan. Notably, *bla*_OXA-23_ is the most prevalent carbapenemase-encoding gene among CRAB worldwide. Studies from Uruguay, Thailand, Tunisia, and Italy also showed that the *bla*_OXA-23-like_ gene was the most prevalent type among CRAB, with prevalences of 77.5%, 81.7%, 85%, and 95%, respectively (18, 63–65). In addition, a recent study analyzed over 1,450 *A*. *baumannii* genomes from more than 40 countries, revealing that the OXA-23 gene family is a significant antibiotic resistance gene, though less prevalent than intrinsic resistance genes, such as efflux pumps and their regulators (66).

Previous studies have shown small proportions of class B carbapenemase or MBL genes among CRA isolates (7, 65, 67). In agreement with these findings, the current study showed that *bla*_IMP_ was only detected in two CR-NAB isolates (strains also carried a *bla*_OXA-58-like_ gene), and *bla*_NDM-1_ was detected in one CR-NAB strain (*A. seifertii*). Although NDM carbapenemases are rarely found in *Acinetobacter* spp., the *bla*_NDM-1_ gene has been sporadically reported in *A. baumannii, A. soli*, *A. pittii, A. nosocomialis*, *A. lwoffii*, and *A. junii*, with isolates originating from both human and environmental isolates across numerous countries, including regions in Asia and the USA (10, 18, 19, 44–47, 68). In this current study, *bla*_NDM-1_ was identified for the first time in *A. seifertii*. This gene, which can be located on plasmids or chromosomes, is transferable between clinical and non-clinical settings, indicating its widespread dissemination (68, 69).

In our present study, we observed significantly higher MICs to carbapenems (IMP and MER) among CRAB isolates carrying *bla*_OXA-24-like_ gene compared with those with *bla*_OXA-23-like_. This aligns with previous findings (61), reporting elevated MICs of carbapenems in OXA-24-like carbapenemase-expressing strains, suggesting that *bla*_OXA-24-like_ imparts greater resistance to carbapenems than *bla*_OXA-23-like_. Generally, class B β-lactamases exhibit stronger carbapenem-hydrolyzing activities compared with class D OXA-type enzymes (7–9); however, we found that the coexistence of *bla*_OXA-58-like_+*bla*_IMP_ did not lead to a high MIC, which could be due to other genetic factors influencing expression or activity levels of IMP carbapenemase. In contrast to the low MICs observed in strains carrying *bla*_IMP_, our study documented significantly elevated MICs of IMP and MER in CRA strains harboring *bla*_NDM-1._ This observation aligns with the characteristic activity of class B β-lactamases, which exhibit broad-spectrum enzymatic activity and resistance to inhibition (15, 16). Therefore, surveillance of its spread among the AB group is worthy of great attention and urgently needed.

The capsule was considered a virulence determinant for CRAB (21). Besides, capsular typing plays a crucial role in clinical *A. baumannii* infection control, as the capsule is both a significant epidemiological marker and the vaccine target (70, 71). Therefore, early detection of capsular type in clinical *A. baumannii* infections is imperative. However, most studies used WGS to detect the KL type. It is difficult to analyze large numbers of clinical isolates using this method due to the expense and complexity of amplifying the entire *cps* region. Based on an investigation by Hu et al., the *wzy* gene, which shows considerable sequence variation across KL types, might be a candidate for a molecular PCR-based serotyping assay (72). We applied *wzy*-based multiplex PCR methods and revealed the predominance of some KL types in our collection. Although certain clones may potentially be circulating within the two hospitals, we observed variability in carbapenemase gene patterns, carbapenem susceptibility (as indicated by MIC values), and ST types, even among isolates sharing the same KL types. This suggests that different clones were included in our collection. Comparing Linkou-CGMH and Kaohsiung-CGMH (178 miles apart), no significant difference in KL-type distribution was observed between the two hospitals, indicating that common KL types exist across different regions in Taiwan. This highlights the significance of the major KL types. Additionally, the multiplex PCR was designed to target KL types commonly reported across different countries or with known clinical significance. For instance, KL2, KL10, KL22, and KL52 have been reported not only in Taiwan but also internationally, underscoring the relevance of these KL types. Therefore, we believe that the *wzy* multiplex PCR method holds significant value for broader applications. The multiplex *wzy* PCR assay is a time- and cost-saving method for *A. baumannii* capsular genotyping, which should facilitate its application. We propose this genotyping method as a useful tool for screening clinically significant KL types of a large number of *A. baumannii* isolates when the use of WGS is impractical. The multiplex *wzy* PCR is anticipated to enhance clinical identification of MDR/CRAB, thereby optimizing treatment strategies and ultimately improving patient outcomes.

Previous studies have documented variants of KL types in *A. baumannii* strains, with KL1 and KL2 as common KL types in GC1 and GC2, respectively (25, 73, 74). A study conducted at a Vietnamese hospital detected various KL types, with KL2 being the most prevalent at 36.4%. A recent study of clinical *A. baumannii* (MDR and drug-susceptible) strains in China showed that KL2 (12.7%) was the most prevalent (75). Cahill et al. analyzed 9,065 genomes from the *Kaptive* database, revealing that KL2 was the predominant type, occurring in 16.5% of genomes, followed by KL3 and KL22 at 13.3% and 6.7%, respectively (35). Most of these findings are consistent with our results, indicating similar trends in the prevalence of KL2 and KL22 in clinical *A. baumannii* strains. However, in our data set, KL3 only accounts for 4.6%. KL81, which closely resembles KL2 except for the presence of *cgm*A, emerged with low prevalences in 2020 and 2021. Further investigation is needed to understand the differential prevalence and pathogenic mechanisms of KL2 and KL81. Besides, KL52 and KL10 accounted for 22.5% and 10% of CRAB isolates, respectively. KL52 and KL10 appear to be geographically restricted, as they have been reported only in Taiwan and Thailand (23); however, a study in Thailand of 191 *A*. *baumannii* isolates revealed more KL10 strains than KL52 strains, with KL52 accounting for only 7.9% of isolates, and KL10 as the most prevalent at 15.7%. In addition, consistent with previous reports from Taiwan and other Asian countries (23, 25, 76, 77), ST2 and ST129 were the most frequently observed sequence types in our collection. Interestingly, however, ST10, ST57, and ST1500, which had not been previously reported in Taiwan, were identified in this study. Notably, among 24 *A*. *seifertii* strains, 15 belonged to ST1500 with the same KL14, indicating that the majority were clonally related, with the isolate carrying *bla*_NDM-1_ (AS39) being one of them, which further acquired the NDM plasmid.

Because NDM-1 is rarely found in *Acinetobacter* spp. in Taiwan, we further investigated the genetic background of the *A. seifertii* isolate carrying *bla*_NDM-1_ (AS39) using WGS. Most acquired antimicrobial resistance genes in AS39 were found on the chromosome, while the carbapenem resistance gene *bla*_NDM-1_ was located on the plasmid pAS39-2 alongside a gene (*aph*(3′)-VI) that conferred resistance to aminoglycosides. An earlier investigation conducted in Taiwan in 2014 showed scattered emergence of NDM-1-positive *A. soli* (harboring pM131, which is almost identical to pAS39-2) (44). Here, we reported NDM-1-positive *A. seifertii* (in 2018) for the first time, suggesting possible transmission of pM131-like plasmids in Taiwan. Notably, the highly similar plasmid pAP_D499 was originally reported in an *A. pittii* isolate from the ICU of a hospital in China in 2008 (45). However, whether pM131 in Taiwan originated from pAP_D499 in China and subsequently evolved into pAS39-2 remains unknown.

Plasmid pM131 was previously shown to be transferred via conjugation. The conjugal transfer efficiency of pM131 into three clinically prominent species (*A. baumannii*, *A. pittii*, and *A. nosocomialis*) was lower than that of other species less commonly associated with human diseases, such as *A. junii*, *A. calcoaceticus*, and *A. soli* (44), suggesting that non-pathogenic *Acinetobacter* species could acquire this plasmid relatively easily and might serve as an environmental reservoir for NDM-1 plasmids. A study conducted in China showed that the *bla*_NDM-1_ plasmid pAP_D499 was prevalent in *A. pittii* and can be transferred to *A. baumannii* via conjugation (45). Both the studies for pM131 and pAP_D499 stated that no transconjugant was obtained when using *E. coli* J53 as a recipient, which is consistent with our findings for pAS39-2. In our tests, pAS39-2 transfer from *A. baumannii* to *A. baumannii* was attempted using an *A. baumannii* transconjugant as the donor, but no successful transfer was achieved. Conjugation efficiency can be affected by the properties of the plasmid, the lipopolysaccharide composition of the outer membrane or inner core of the recipient, certain environmental and media conditions (78). A recent paper reported that bacterial conjugation is very active *in vivo*, especially in the intestinal environment (79). Thus, although no successful transfer of pAS39-2 within *A. baumannii* strains were detected under our experimental conditions, we cannot exclude that conjugative transfer could occur *in vitro* under different conditions or *in vivo*.

## MATERIALS AND METHODS

### Study population

Isolates of the *A. baumannii* group were collected at two tertiary hospitals: Chang Gung Memorial Hospital (CGMH)-Lin Kou branch, a 3,700-bed medical center in northern Taiwan, and CGMH-Kaohsiung branch, a 2,700-bed medical center in southern Taiwan, from January 2015 to October 2021. The iterative process of sample collection was described as follows. A total of 1,265 samples were collected, non-bacteremia samples (*n* = 734) were excluded, and repetitive samples from the same patient (*n* = 39) were excluded. Finally, a total of 492 samples from patients with bacteremia (either nosocomial or community-acquired) were analyzed in this study (Fig. 1A; Table S1). The *Acinetobacter* species were identified using MALDI-TOF MS (29, 30) and *bla*_OXA-51-like_ PCR (30–32). Isolates with a MIC of IMP and MER >4 mg/L using the broth dilution method according to 2021 Clinical and Laboratory Standards Institute interpretive criteria for *Acinetobacter* spp. were considered carbapenem-resistant strain (80).

### Carbapenemase genes

Multiplex PCR for *bla*_OXA-23-like_, *bla*_OXA-24-like_, *bla*_OXA-51-like_, *bla*_OXA-58-like_, *bla*_OXA-143-like_, *bla*_KPC_, *bla*_GES_, *bla*_NDM_, *bla*_IMP_, *bla*_VIM_, *bla*_SPM_, *bla*_GIM_, and *bla*_SIM_ was performed following a previously reported protocol (58, 81, 82), with minor modifications. The preparation of the bacterial template and PCR conditions are detailed in the Supplementary Methods. For CSA strains not subjected to multiplex carbapenemase PCR, a singleplex PCR using OXA51-like primers was performed to detect the presence of a *bla*_OXA-51-like_ gene. The PCR conditions were the same, except that an annealing temperature of 56°C was used.

### Comparative sequence analysis of the *rpo*B gene

The *rpo*B types of 239 *A*. *baumannii* and 27 NAB strains were identified using the Pasteur scheme MLST database. The primers and PCR conditions were as previously described (1, 33). PCR amplicons were subjected to Sanger sequencing and were analyzed using the PubMLST website for *rpo*B allele determination.

### Multiplex *wzy* PCR assay

Eleven primer pairs designed to detect *wzy* of types KL1, KL2/KL81, KL3/KL22, KL6, KL9, KL10, KL14, KL47, KL49, and KL52, and *cgm*A were divided into three stages (M1, M2, and M3) (Fig. 3A) and were used to detect KL type-specific *wzy* genes, with product sizes of 259–764 bp (Fig. 3B), including six primer pairs used in a previous study (22) and five newly designed primer pairs (Table 1). The preparation of the bacterial template and PCR conditions are detailed in the Supplemental Methods.

### Bacterial genome sequencing and sequence analyses

The DNA extraction of NDM-positive NAB strain (AS39) and subsequent sequencing were conducted following the detailed methods outlined in the Supplemental Methods section.

### Conjugation assay

A conjugation assay to assess the transmissibility of pAS39-2 from its natural CR-NAB host, AS39, to the clinical *A. baumannii* isolates was performed as described previously (51, 83), with some modifications. The detailed methods are described in the Supplemental Methods. The MICs for the successful transconjugants were also determined (Table 4).

### Plasmid stability

Plasmid stability was assessed as previously described, with minor modifications (84). The detailed methods are described in the Supplemental Methods.

## SUPPLEMENTAL MATERIAL

Table S1 msphere.00793-24-s0001.xlsx Information on the isolates used in this study.

Supplemental Figures msphere.00793-24-s0002.docx Figures S1 to S6.

Supplemental Methods msphere.00793-24-s0003.docx Details of bacterial template and PCR, MLST, bacterial genome sequencing, conjugation assay, and plasmid stability testing.

Additional Supplemental Tables msphere.00793-24-s0004.docx Tables S2 to S9.

ASM does not own the copyrights to Supplemental Material that may be linked to, or accessed through, an article. The authors have granted ASM a non-exclusive, world-wide license to publish the Supplemental Material files. Please contact the corresponding author directly for reuse.
