# Supplementary material for: Molecular epidemiology of carbapenem-resistant Acinetobacter baumannii group in Taiwan
Source: mSphere. 2024 Dec 31;10(1):e00793-24. doi: 10.1128/msphere.00793-24 (PMC11774041; doi:10.1128/msphere.00793-24)
Supplement: Supplemental Figures — Figures S1 to S6. [file msphere.00793-24-s0002.docx]

**
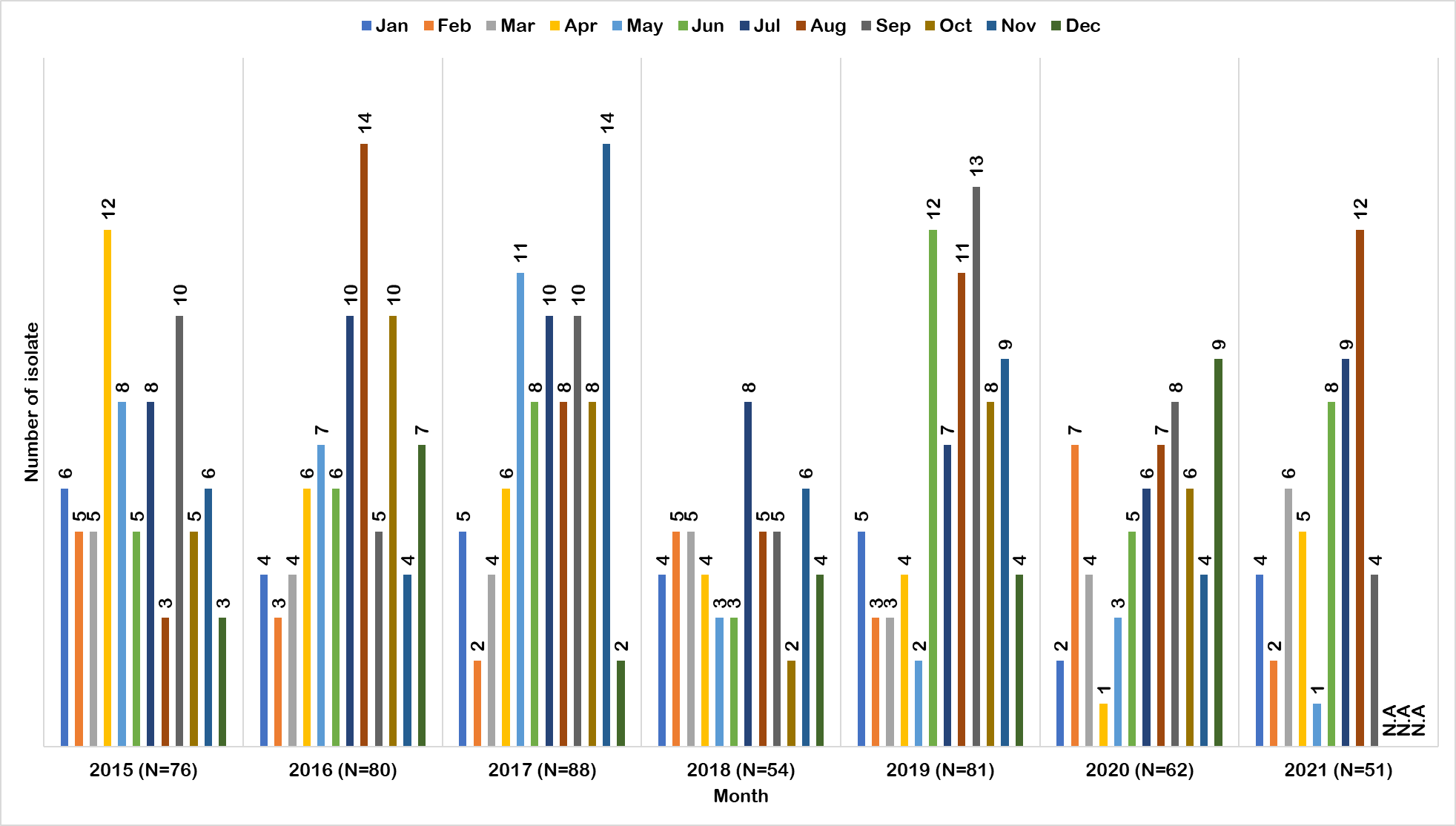
Supplemental Figures**

**FIG S1.** The distribution of the 492 isolates across twelve months over a seven-year period. N.A., Not Applicable

**
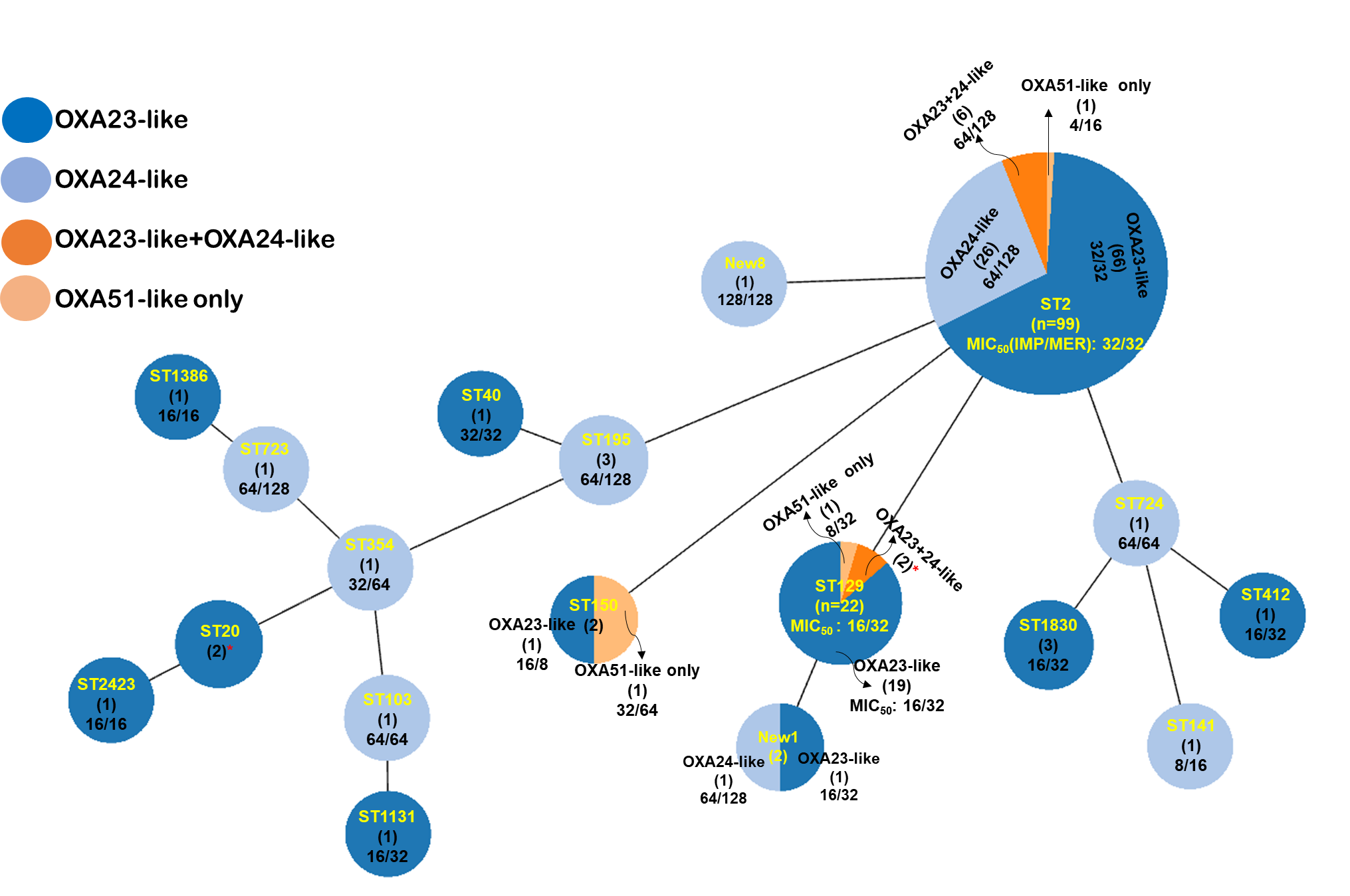
(A)**

**FIG S2.** ST-Based Clustering of *A. baumannii* Strains. (A) Distribution of OXA Types within ST Clusters of 144 CRAB strains with identified ST types; Minimum Inhibitory Concentration (MIC) of imipenem and meropenem were shown as by (IMP/MER): MIC_50_, which represents the minimum inhibitory concentration required to inhibit the growth of 50% of the bacterial population was calculated for different ST types or different OXA types within the ST type; however, for ST types with only two isolates where MIC_50_ was not calculated (marked with an asterisk), the IMP/MER MIC values are as follows: ST129-OXA_23+24_like (64/128 and 128/64), ST20 (16/32 and 32/32). For ST types with a single isolate, the MIC values for that strain are shown directly. The number of isolates is displayed in parentheses. The alleles of new ST types (New1, New8) are listed in Table S1.

**
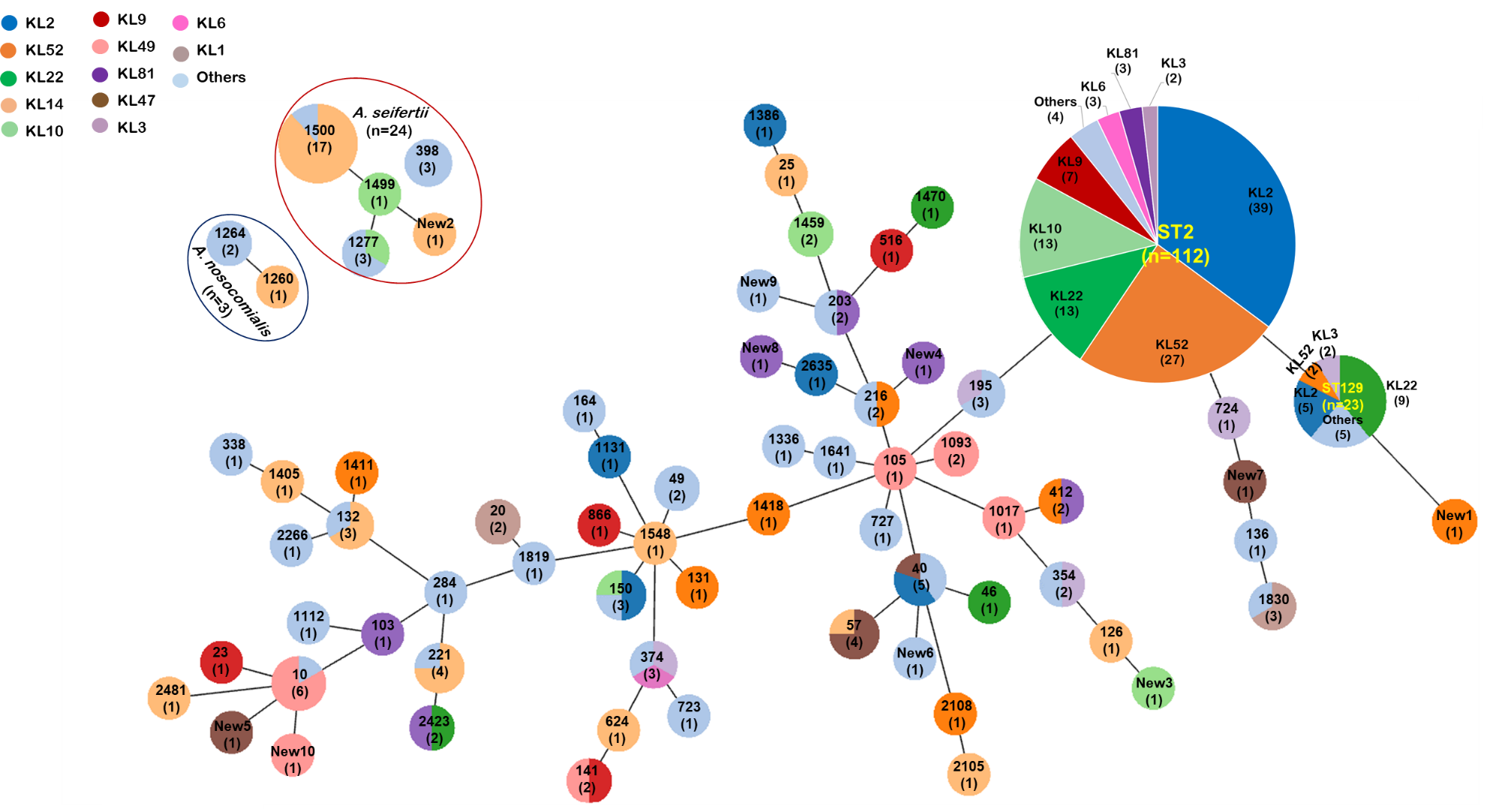
(B)**

**FIG S2.** Continued. (B) Distribution of KL Types within ST Clusters of 262 AB-group strains (235 *A. baumannii*, 24 *A. seifertii*, 3 *A. nosocomialis*) with identified ST types. The red circle groups the 24 strains with *rpoB* matching to *A. seifertii*, while the blue circle includes three strains with *rpoB* matching to *A. nosocomialis*. The number of isolates is displayed in parentheses. The alleles of new ST types (New1 – New10) are listed in Table S1.

**
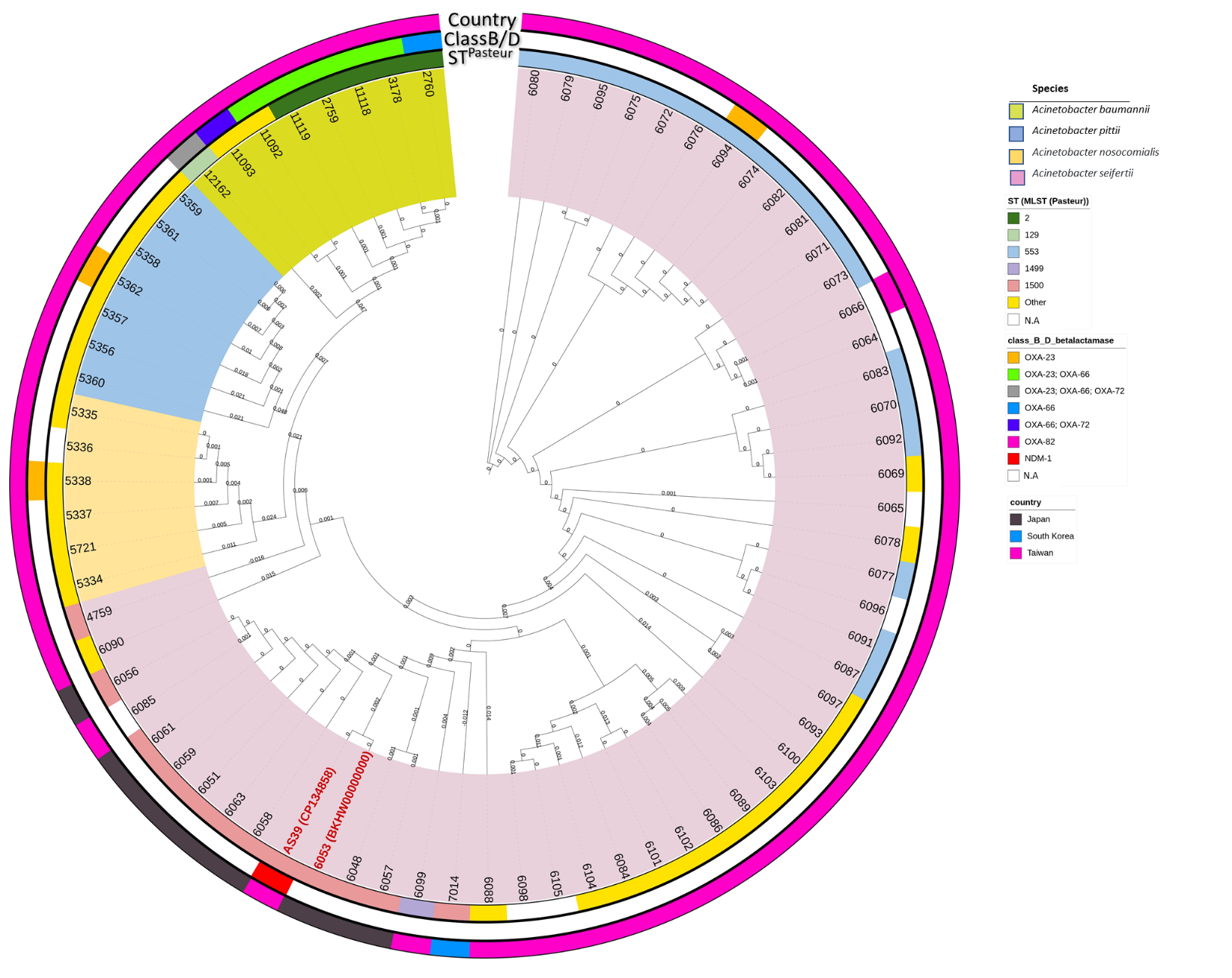
**

**FIG S3.** Phylogenetic trees of *A. seifertti* AS39 in comparison with other related isolates


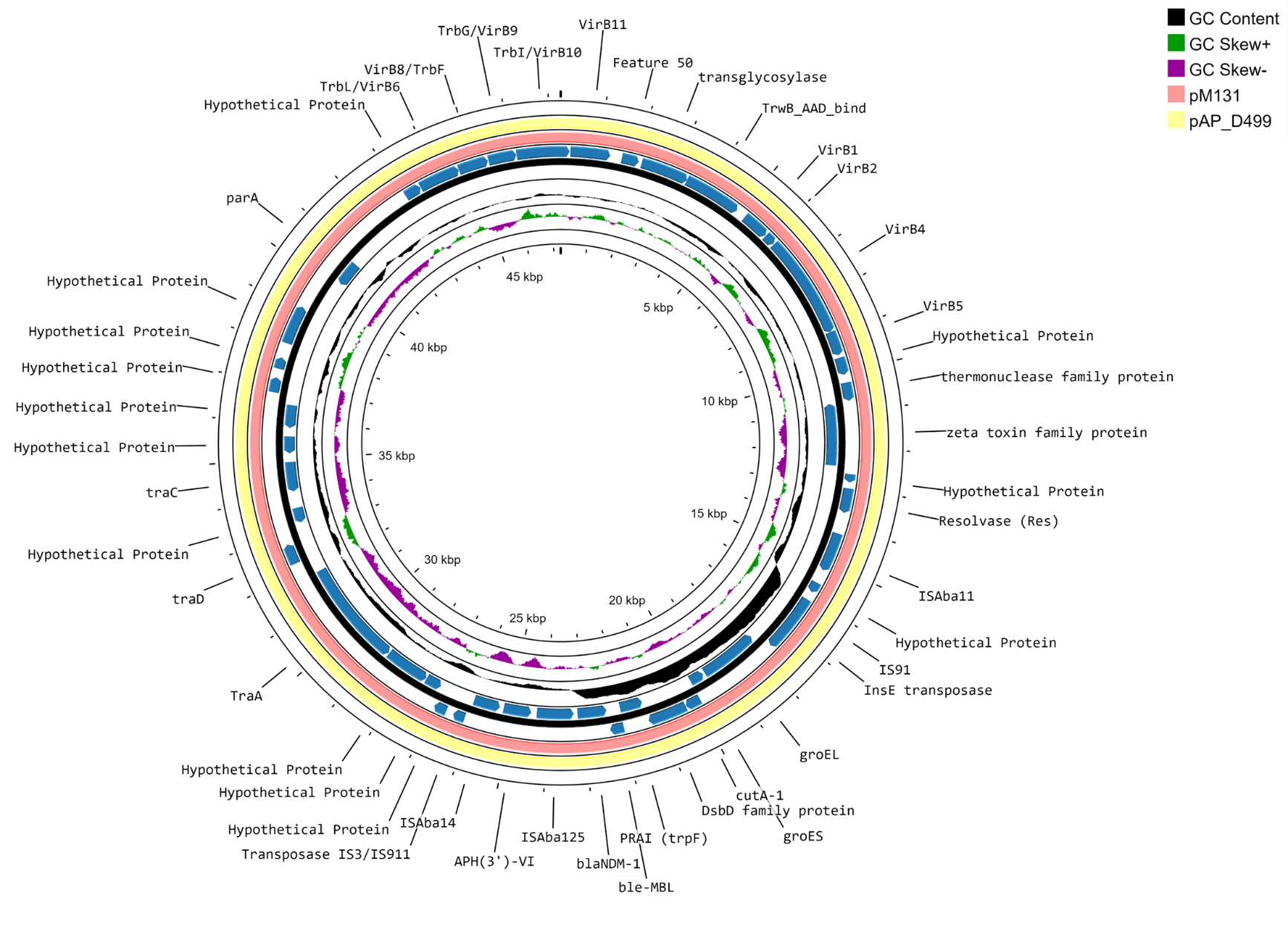


**FIG S4.** Comparison of pAS39-2 with plasmids pM131 from *A. soli* M131 and pAP_D499 from *A. pittii* AP_D499 strain

**(A) (B)**

**
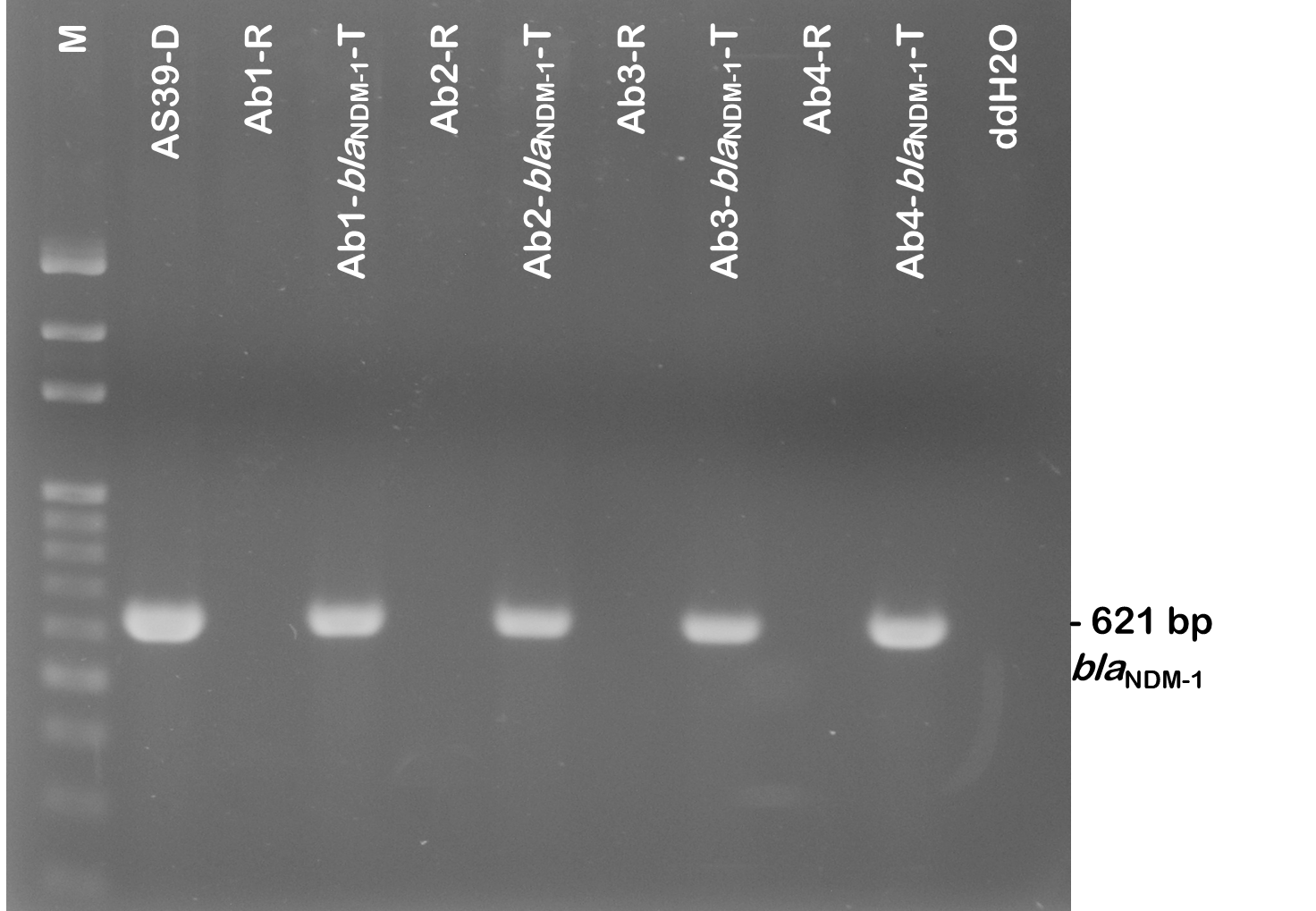

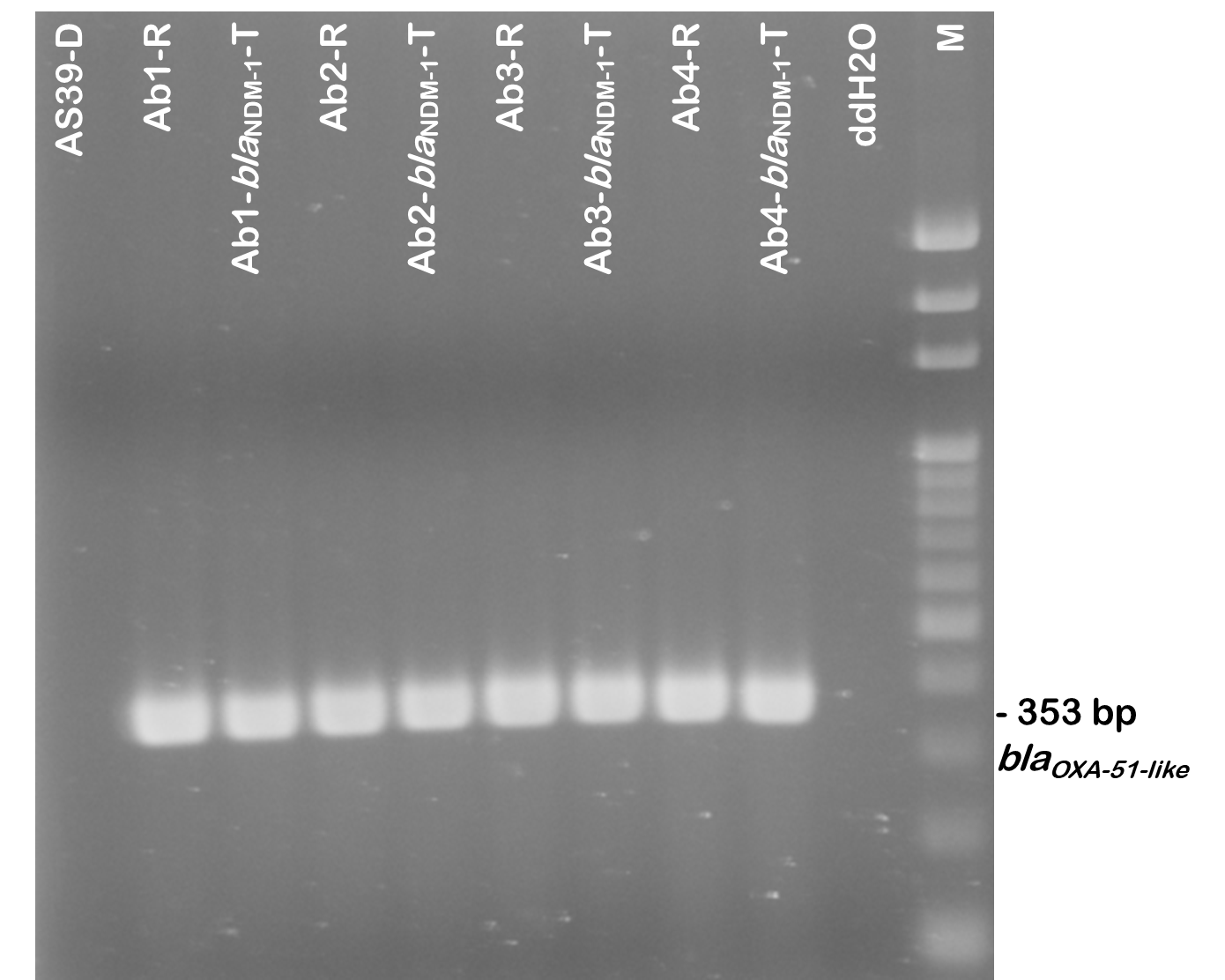
**

**
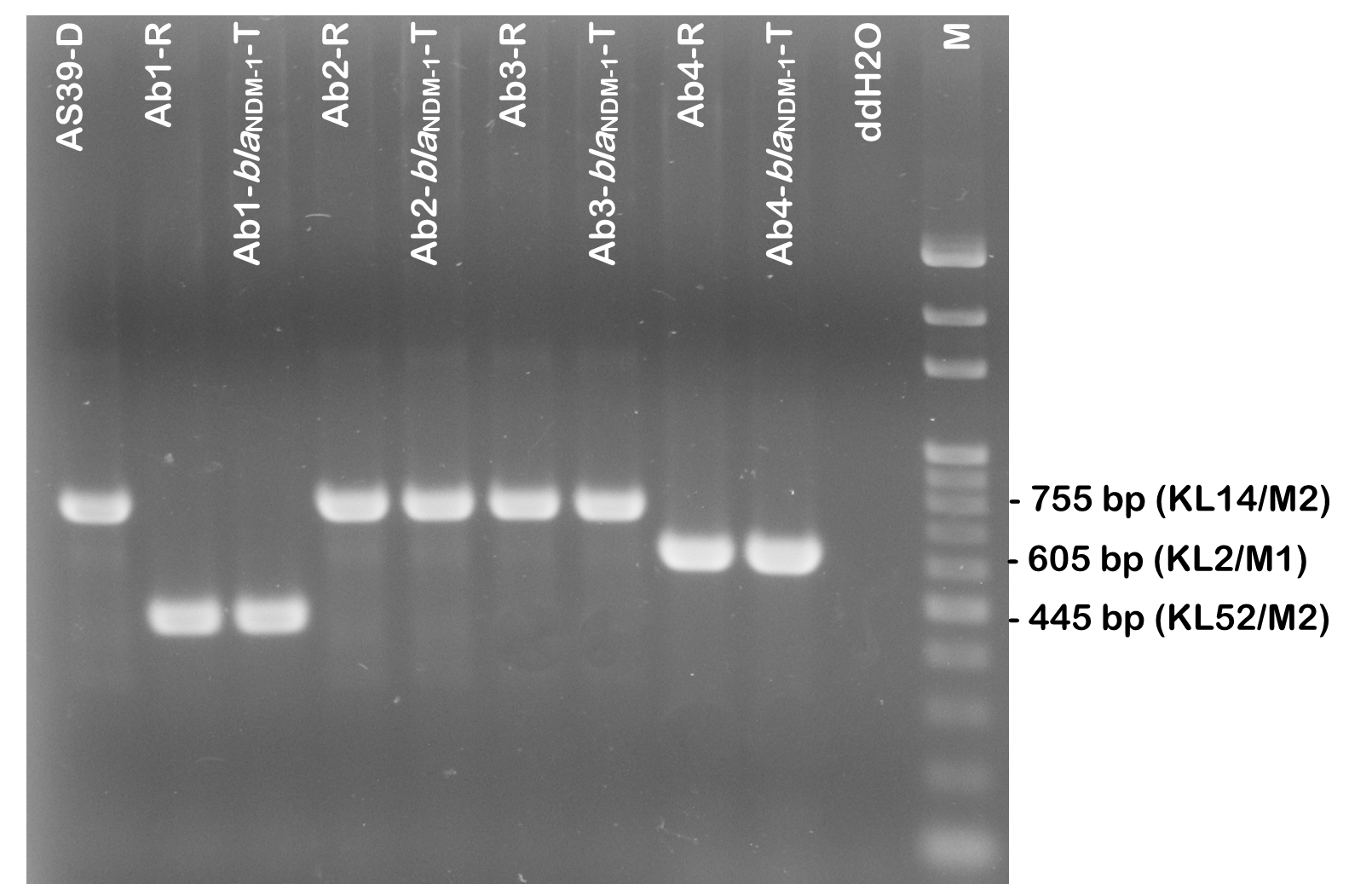
(C)**

**FIG S5.** Differentiation and confirmation of donor, recipients, and transconjugants

**(A)**

**
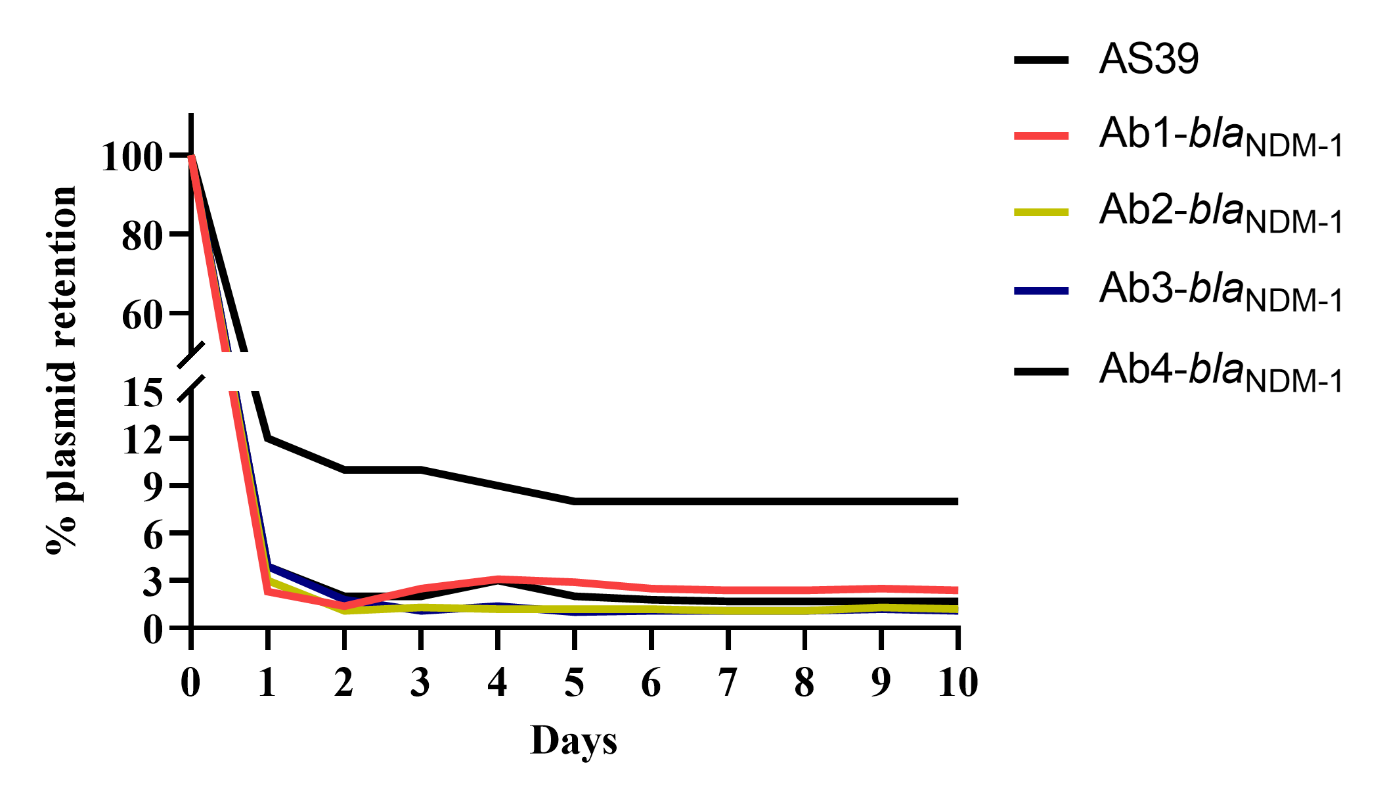
**

**
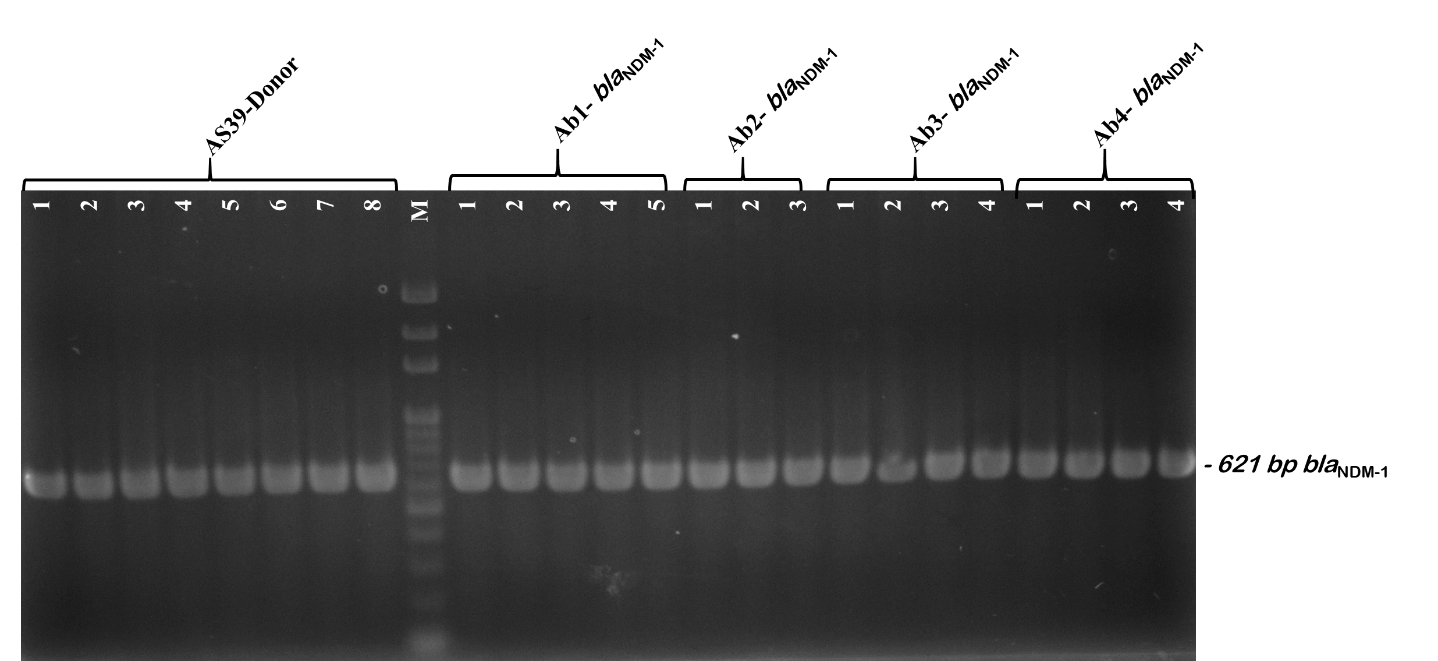
(B)**

**FIG S6.** Stability of *bla*_NDM-1_ plasmids along 10-day serial passage
