# Supplementary material for: Molecular epidemiology of carbapenem-resistant Acinetobacter baumannii group in Taiwan
Source: mSphere. 2024 Dec 31;10(1):e00793-24. doi: 10.1128/msphere.00793-24 (PMC11774041; doi:10.1128/msphere.00793-24)
Supplement: Supplemental Methods — Details of bacterial template and PCR, MLST, bacterial genome sequencing, conjugation assay, and plasmid stability testing. [file msphere.00793-24-s0003.docx]

**Supplemental Methods:**

**Bacterial template and** **polymerase chain reaction (PCR).** Overnight bacterial cultures were heated at 96℃ for 15 min and used as a template. For each reaction, 3 μL of the bacterial suspension was added to a 17 µL mixture containing 2 µL of 10× DreamTaq buffer, 0.2 µL of DreamTaq polymerase, 2 µL of 10 mM deoxynucleoside triphosphates (dNTPs) mix (2.5 mM each), 0.5 µL of each primer (10 µM), 1 µL of 5% dimethyl sulfoxide, and 7.8 µL of ddH2O.

PCR was conducted under the following cycling conditions: 96℃ for 3 min, followed by 30 cycles of amplification consisting of 96℃ for 30 s, 54℃ for 30 s, and 72℃ for 1 min, with a final extension for 10 min at 72℃. The products were loaded in a 1.7% agarose gel, separated by electrophoresis at 150 V/400 mA for 22 min with 1× TAE buffer (40 mM Tris-acetate and 1 mM EDTA, pH 8.0) and then stained with ethidium bromide (EtBr).

**Multi-locus sequence type (MLST).** Sequence types (STs) of 235 *A. baumannii* (144 CRAB and 91 CSAB) and all 27 non-AB strains were identified using the Pasteur scheme for MLST. Among the 235 *A. baumannii* strains, all eight strains co-harboring *bla*_OXA-23-like_ and *bla*_OXA-24-like_ genes were included, regardless of their KL type; For KL2, 49 strains with either *bla*_OXA-23-like_, *bla*_OXA-24-like_, or *bla*_OXA-51-like_ only were randomly selected from 68 strains; Similarly, we selected representative isolates from other major KL types: 35/63 KL52 strains, 22/44 KL22, 17/32 KL10, and 14/25 KL14 strains; Less prevalent KL types that emerged over several years were also included, such as KL1 (4/4), KL3 (8/11), KL6 (4/5), KL9 (10/19), KL47 (7/12), KL49 (11/13), and KL81 (9/12); Also, other types were randomly selected including CRAB (12/15) and CSAB (25/128). Primers were obtained from the PubMLST website (https://pubmlst.org/primers-used-mlst-acinetobacter-baumannii-complex-pasteur-scheme). PCR amplicons were subjected to Sanger sequencing, and ST determination was performed using the PubMLST database.

**Bacterial genome sequencing**

Genomic DNA of the NDM-positive NAB strain (AS39) was extracted using QIAamp PowerFecal Pro DNA Kit (Qiagen) following a size selection by using the Short Read Eliminator XS (PacBio) to deplete DNA fragments < 10kb. DNA concentration was determined by Qubit 4.0 fluorometer (Thermo Scientific) and the fragment size was monitored by the Qsep 100TM system (Taiwan). Long-read sequencing libraries were constructed through end-repair, A-tailing, barcoding (e.g., Multiplexing), and adapter ligation. The sequencing results were verified using NanoPlot to validate the read length profile. Next, the raw reads were assembled into primary contigs using Flye (1)/Canu (2). The primary contigs were then polished through model correction using Medaka (3). The contigs were also corrected based on homologous sequences extracted from closely related genomes using Homopolish (4). The quality of the fully polished contigs was assessed using QUAST (5), and the completeness of the genome was evaluated using BUSCO (6). PROKKA, with default settings, was used to predict open reading frames (ORFs) and locate tRNA and rRNA regions (7). Gene annotation was performed by aligning the sequences against the RefSeq, COG, eggNOG, KOfam, VFDB, CARD, NCyc, dbCAN2, BacMet, and PHIBase databases using DIAMOND, HMMER, and database-specific annotators (8-16).

Two schemes, the Housekeeping genes of Pasteur (17) and Oxford (18), were used for ST assignment along with the MLST database (19). The comparative genome analysis of *Acinetobacter* species included published *Acinetobacter* genomes from Taiwan as well as strains belonging to ST1500 and single locus variant of ST1500 (ST1499, ST-new) from other countries. The resulting *Acinetobacter* species phylogenetic trees were visualized using iTOL v5 (<https://itol.embl.de/>). SnapGene 3.2.1 was used to visualize and manipulate the sequences. Comparative analysis of DNA identity was performed using Easyfig 2.2.2 (20). Plasmids identical to pAS39-2 in the nonredundant (nr) nucleotide collection were searched using BLASTN, and the search was initially optimized for highly similar sequences (MegaBLAST) (<https://blast.ncbi.nlm.nih.gov/Blast.cgi>). Antibiotic resistance genes were identified using CARD (<https://card.mcmaster.ca/>) and ResFinder (<https://cge.cbs.dtu.dk/services/ResFinder/>). Then, ISFinder (<https://isfinder.biotoul.fr/>) was used to identify insertion sequences. SeCreT4 (<https://db-mml.sjtu.edu.cn/SecReT4/>) was used to identify putative conjugative transfer genes.

**Conjugation assay.** As a positive control to ensure that the experimental procedures were successful, we used a donor, carbapenem-resistant *K. pneumoniae* 17CRE24 (collected from Tung’s Taichung MetroHarbor Hospital, Taiwan), which was able to transfer the *bla*_OXA-48_ gene to sodium azide-resistant (SAR) *E. coli* J53 (recipient) by conjugation. Since AS39 was susceptible to chloramphenicol, as recipients, we randomly selected 20 *A. baumannii* strains that showed carbapenem susceptibility but chloramphenicol resistance. Either AS39 or 17CRE24 was used as a donor, and either *E. coli* J53 or a chloramphenicol-resistant *A. baumannii* (ChR-AB) isolate was used as the recipient. The donors were cultured overnight at 37°C in LB broth supplemented with 4 mg/L imipenem, and the recipients were cultured in LB broth supplemented with 100 mg/L SAZ (for J53) or 50 mg/L chloramphenicol (for ChR-AB). The donor and recipient cells were mixed at 1:5 (200 µL:1 mL) and centrifuged at 4,000 🞨 g for 5 min. The cell pellet was resuspended in ~2 µL of LB broth, and the cell suspension was incubated for 1 h before transferring to a nitrocellulose membrane on an LB agar plate and incubating overnight. The nitrocellulose membrane was then transferred to a tube containing fresh LB broth and incubated at 37°C for 30 min with shaking. Transconjugants were selected on LB agar containing 100 mg/L SAZ or 50 mg/L chloramphenicol supplemented with 4 mg/L imipenem. Successful transfer of the plasmid was confirmed via PCR to detect *bla*_NDM-1_, *bla*_OXA-51-like_, and multiplex *wzy*, which differentiates the donor and recipient based on capsular type (FIG S5). *E. coli* J53 transconjugants were also plated on eosin methylene blue agar to confirm their identity as *E. coli*, which produces colonies with a green metallic sheen on this medium.

**Plasmid stability testing.** Cultures were grown at 37°C in a shaking incubator and serially passaged for 10 days at a 1:1000 dilution in antibiotic-free LB broth. Cultures were serially diluted daily and plated on both antibiotic-free LB agar and LB agar containing 4 mg/L imipenem. The retention rate of the NDM-1 plasmid was calculated by dividing the number of colonies on the imipenem-containing LB agar plate by the number of colonies on the antibiotic-free LB agar plate. On day 10, colonies were randomly selected and subjected to PCR to detect *bla*_NDM-1_ for confirmation (FIG S6).

**Supplemental References**

1. Kolmogorov M, Yuan J, Lin Y, Pevzner PA. 2019. Assembly of long, error-prone reads using repeat graphs. Nat Biotechnol 37:540-546.

2. Koren S, Walenz BP, Berlin K, Miller JR, Bergman NH, Phillippy AM. 2017. Canu: scalable and accurate long-read assembly via adaptive k-mer weighting and repeat separation. Genome Res 27:722-736.

3. Vaser R, Sovic I, Nagarajan N, Sikic M. 2017. Fast and accurate de novo genome assembly from long uncorrected reads. Genome Res 27:737-746.

4. Huang YT, Liu PY, Shih PW. 2021. Homopolish: a method for the removal of systematic errors in nanopore sequencing by homologous polishing. Genome Biol 22:95.

5. Gurevich A, Saveliev V, Vyahhi N, Tesler G. 2013. QUAST: a quality assessment tool for genome assemblies. Bioinformatics 29:1072–1075.

6. Simao FA, Waterhouse RM, Ioannidis P, Kriventseva EV, Zdobnov EM. 2015. BUSCO: assessing genome assembly and annotation completeness with single-copy orthologs. Bioinformatics 31:3210–3212.

7. Seemann T. 2014. Prokka: rapid prokaryotic genome annotation. Bioinformatics 30:2068–2069.

8. Roman L. Tatusov MYG, Darren A. Natale and Eugene V. Koonin. 2000. The COG database: a tool for genome-scale analysis of protein functions and evolution. Nucleic Acids Research 28:33–36.

9. Chen L, Yang J, Yu J, Yao Z, Sun L, Shen Y, Jin Q. 2005. VFDB: a reference database for bacterial virulence factors. Nucleic Acids Res 33:D325–D328.

10. Urban M, Cuzick A, Seager J, Wood V, Rutherford K, Venkatesh SY, De Silva N, Martinez MC, Pedro H, Yates AD, Hassani-Pak K, Hammond-Kosack KE. 2020. PHI-base: the pathogen-host interactions database. Nucleic Acids Res 48:D613-D620.

11. Tu Q, Lin L, Cheng L, Deng Y, He Z. 2019. NCycDB: a curated integrative database for fast and accurate metagenomic profiling of nitrogen cycling genes. Bioinformatics 35:1040-1048.

12. Pruitt KD, Tatusova T, Maglott DR. 2007. NCBI reference sequences (RefSeq): a curated non-redundant sequence database of genomes, transcripts, and proteins. Nucleic Acids Res 35:D61–D65.

13. Huerta-Cepas J, Szklarczyk D, Forslund K, Cook H, Heller D, Walter MC, Rattei T, Mende DR, Sunagawa S, Kuhn M, Jensen LJ, von Mering C, Bork P. 2016. eggNOG 4.5: a hierarchical orthology framework with improved functional annotations for eukaryotic, prokaryotic, and viral sequences. Nucleic Acids Res 44:D286–D293.

14. Zhang H, Yohe T, Huang L, Entwistle S, Wu P, Yang Z, Busk PK, Xu Y, Yin Y. 2018. dbCAN2: a meta server for automated carbohydrate-active enzyme annotation. Nucleic Acids Res 46:W95-W101.

15. Pal C, Bengtsson-Palme, J., Rensing, C., Kristiansson, E., Larsson, DGJ. 2014. BacMet: antibacterial biocide and metal resistance genes database. Nucleic Acids Res 43:D737-D743.

16. Jia B, Raphenya AR, Alcock B, Waglechner N, Guo P, Tsang KK, Lago BA, Dave BM, Pereira S, Sharma AN, Doshi S, Courtot M, Lo R, Williams LE, Frye JG, Elsayegh T, Sardar D, Westman EL, Pawlowski AC, Johnson TA, Brinkman FS, Wright GD, McArthur AG. 2017. CARD 2017: expansion and model-centric curation of the comprehensive antibiotic resistance database. Nucleic Acids Res 45:D566-D573.

17. Diancourt L, Passet V, Nemec A, Dijkshoorn L, Brisse S. 2010. The population structure of *Acinetobacter baumannii*: expanding multiresistant clones from an ancestral susceptible genetic pool. PLoS One 5:e10034.

18. Bartual SG, Seifert H, Hippler C, Luzon MA, Wisplinghoff H, Rodriguez-Valera F. 2005. Development of a multilocus sequence typing scheme for characterization of clinical isolates of *Acinetobacter baumannii*. J Clin Microbiol 43:4382–4390.

19. Jolley KA, Bray JE, Maiden MCJ. 2018. Open-access bacterial population genomics: BIGSdb software, the PubMLST.org website, and their applications. Wellcome Open Res 3:124.

20. Sullivan MJ, Petty NK, Beatson SA. 2011. Easyfig: a genome comparison visualizer. Bioinformatics 27:1009–1010.
