# Supplementary material for: Molecular epidemiology of carbapenem-resistant Acinetobacter baumannii group in Taiwan
Source: mSphere. 2024 Dec 31;10(1):e00793-24. doi: 10.1128/msphere.00793-24 (PMC11774041; doi:10.1128/msphere.00793-24)
Supplement: Additional Supplemental Tables — Tables S2 to S9. [file msphere.00793-24-s0004.docx]

**Supplemental Tables**

**Table S2** The distribution of KL types by carbapenemase genes (*p*-value = 0.0005 for Fisher exact test)

|  | **Carbapenemase gene pattern** | | | |
| --- | --- | --- | --- | --- |
|  | Oxa23-like (N=172) | Oxa24-like  (N=54) | Oxa23-like+Oxa24-like (N=8) | Other^a^ (N=6) |
| **KL type** | n (%) | n (%) | n (%) | n (%) |
| KL1 | 4 (2.33) | 0 (0) | 0 (0) | 0 (0) |
| KL2 | 39 (22.67) | 26 (48.15) | 2 (25) | 1 (16.67) |
| KL3 | 5 (2.91) | 4 (7.41) | 0 (0) | 0 (0) |
| KL6 | 3 (1.74) | 1 (1.85) | 0 (0) | 0 (0) |
| KL9 | 3 (174) | 6 (11.11) | 1 (12.5) | 0 (0) |
| KL10 | 19 (11.05) | 5 (9.26) | 0 (0) | 1 (16.67) |
| KL14 | 0 (0) | 0 (0) | 0 (0) | 1 (16.67) |
| KL22 | 34 (19.77) | 0 (0) | 3 (37.5) | 1 (16.67) |
| KL47 | 0 (0) | 2 (3.7) | 0 (0) | 0 (0) |
| KL49 | 0 (0) | 1 (1.85) | 0 (0) | 0 (0) |
| KL52 | 49 (28.49) | 3 (5.56) | 2 (25) | 0 (0) |
| KL81 | 5 (2.91) | 3 (5.56) | 0 (0) | 0 (0) |
| Other^b^ | 11 (6.4) | 3 (5.56) | 0 (0) | 2 (33.33) |

^a^: *bla*_NDM-1_ (n=1): KL14; *bla*_OXA-58_+*bla*_IMP_ (n=2): KL10 and other; *bla*_OXA-51-like_ only (n=3): KL22, KL2, and other

^b^: non-KL1/2/3/6/9/10/14/22/47/49/52/81 (non-M1/2/3)

Note: 240 CRA isolates with carbapenemase genes are included in this table.

**Table S3** The distribution of ST types by carbapenemase genes (*p*-value = 0.0005 for Fisher exact test )

|  | **Carbepenemase gene pattern** | | | |
| --- | --- | --- | --- | --- |
|  | Oxa23-like (N=97) | Oxa24-like (N=36) | Oxa23-like + Oxa24-like (N=8) | Other^a^ (N=6) |
| **ST type** | n (%) | n (%) | n (%) | n (%) |
| 2 | 66 (68.04) | 26 (72.22) | 6 (75) | 1 (16.67) |
| 10 | 0 (0) | 0 (0) | 0 (0) | 0 (0) |
| 40 | 1 (1.03) | 0 (0) | 0 (0) | 0 (0) |
| 57 | 0 (0) | 0 (0) | 0 (0) | 0 (0) |
| 129 | 19 (19.59) | 0 (0) | 2 (25) | 1 (16.67) |
| 132 | 0 (0) | 0 (0) | 0 (0) | 0 (0) |
| 150 | 1 (1.03) | 0 (0) | 0 (0) | 1 (16.67) |
| 195 | 0 (0) | 3 (8.33) | 0 (0) | 0 (0) |
| 221 | 0 (0) | 0 (0) | 0 (0) | 0 (0) |
| 374 | 0 (0) | 0 (0) | 0 (0) | 0 (0) |
| 1500 | 0 (0) | 0 (0) | 0 (0) | 1 (16.67) |
| 1830 | 3 (3.09) | 0 (0) | 0 (0) | 0 (0) |
| New^b^ | 1 (1.03) | 2 (5.56) | 0 (0) | 0 (0) |
| Other^c^ | 6 (6.19) | 5 (13.89) | 0 (0) | 2 (33.33) |

^a^: *bla*_NDM-1_ (n=1): ST1500; *bla*_OXA-58_+*bla*_IMP_ (n=2): ST1499, other; *bla*_OXA-51-like_ only (n=3): ST2, ST129, and ST150

^b^: unidentified ST types using the Pasteur database are designated as new.

^c^: Strains with an identified ST type but fewer than two isolates were grouped as ‘other’.

Note: 147 CRA isolates with carbapenemase genes and subjected to ST typing are included in this table.

**Table S4** The distribution of ST types by KL types (*p*-value = 0.0005 for Fisher exact test)

|  | **KL type** | | | | | | | | | | | | |
| --- | --- | --- | --- | --- | --- | --- | --- | --- | --- | --- | --- | --- | --- |
|  | KL1 (N=4) | KL2 (N=51) | KL3 (N=8) | KL6 (N=4) | KL9 (N=11) | KL10 (N=19) | KL14 (N=30) | KL22 (N=25) | KL47 (N=7) | KL49 (N=11) | KL52 (N=37) | KL81 (N=9) | Other^a^  (N=46) |
| **ST type** | n (%) | n (%) | n (%) | n (%) | n (%) | n (%) | n (%) | n (%) | n (%) | n (%) | n (%) | n (%) | n (%) |
| 2 | 0 (0) | 39 (76.5) | 2 (25) | 3 (75) | 7 (63.6) | 13 (68.4) | 0 (0) | 13 (52) | 1 (14.3) | 0 (0) | 27 (73) | 3 (33.3) | 4 (8.7) |
| 10 | 0 (0) | 0 (0) | 0 (0) | 0 (0) | 0 (0) | 0 (0) | 0 (0) | 0 (0) | 0 (0) | 5 (45.5) | 0 (0) | 0 (0) | 1 (2.17) |
| 40 | 0 (0) | 2 (3.9) | 0 (0) | 0 (0) | 0 (0) | 0 (0) | 0 (0) | 0 (0) | 1 (14.3) | 0 (0) | 0 (0) | 0 (0) | 2 (4.35) |
| 57 | 0 (0) | 0 (0) | 0 (0) | 0 (0) | 0 (0) | 0 (0) | 1 (3.3) | 0 (0) | 3 (42.9) | 0 (0) | 0 (0) | 0 (0) | 0 (0) |
| 129 | 0 (0) | 5 (9.8) | 2 (25) | 0 (0) | 0 (0) | 0 (0) | 0 (0) | 9 (36) | 0 (0) | 0 (0) | 2 (5.4) | 0 (0) | 5 (10.9) |
| 132 | 0 (0) | 0 (0) | 0 (0) | 0 (0) | 0 (0) | 0 (0) | 2 (6.7) | 0 (0) | 0 (0) | 0 (0) | 0 (0) | 0 (0) | 1 (2.17) |
| 150 | 0 (0) | 2 (3.9) | 0 (0) | 0 (0) | 0 (0) | 1 (5.26) | 0 (0) | 0 (0) | 0 (0) | 0 (0) | 0 (0) | 0 (0) | 1 (2.17) |
| 195 | 0 (0) | 0 (0) | 1 (12.5) | 0 (0) | 0 (0) | 0 (0) | 0 (0) | 0 (0) | 0 (0) | 0 (0) | 0 (0) | 0 (0) | 2 (4.35) |
| 221 | 0 (0) | 0 (0) | 0 (0) | 0 (0) | 0 (0) | 0 (0) | 3 (10) | 0 (0) | 0 (0) | 0 (0) | 0 (0) | 0 (0) | 1 (2.17) |
| 374 | 0 (0) | 0 (0) | 1 (12.5) | 1 (25) | 0 (0) | 0 (0) | 0 (0) | 0 (0) | 0 (0) | 0 (0) | 0 (0) | 0 (0) | 1 (2.17) |
| 1500 | 0 (0) | 0 (0) | 0 (0) | 0 (0) | 0 (0) | 0 (0) | 15 (50) | 0 (0) | 0 (0) | 0 (0) | 0 (0) | 0 (0) | 2 (4.35) |
| 1830 | 2 (50) | 0 (0) | 0 (0) | 0 (0) | 0 (0) | 0 (0) | 0 (0) | 0 (0) | 0 (0) | 0 (0) | 0 (0) | 0 (0) | 1 (2.17) |
| New^b^ | 0 (0) | 0 (0) | 0 (0) | 0 (0) | 0 (0) | 1 (5.26) | 1 (3.3) | 0 (0) | 2 (28.6) | 1 (9.09) | 2 (5.4) | 2 (22.2) | 2 (4.35) |
| Other^c^ | 2 (50) | 3 (5.9) | 2 (25) | 0 (0) | 4 (36.4) | 4 (21.1) | 8 (26.7) | 3 (12) | 0 (0) | 5 (45.5) | 6 (22.2) | 4 (44.5) | 23 (50) |

^a^: non-KL1/2/3/6/9/10/14/22/47/49/52/81 (non-M1/2/3)

^b^: unidentified ST types using the Pasteur database are designated as new.

^c^: Strains with an identified ST type but fewer than two isolates were grouped as ‘other’.

Note: a total of 262 isolates including 147 carbapenem-resistant (CRA) and 115 carbapenem-susceptible isolates (CSA) subjected to ST typing are included in this table.

**Table S5** Clinical Characteristics of Patients with *Acinetobacter baumannii* group Bacteremia (*p*-value for Fisher exact test or ANOVA test)

|  | **KL type** | | | | | | | | | | | | | |  |
| --- | --- | --- | --- | --- | --- | --- | --- | --- | --- | --- | --- | --- | --- | --- | --- |
|  | KL1 | KL2 | KL3 | KL6 | KL9 | KL10 | KL14 | KL22 | KL47 | KL49 | KL52 | KL81 | Other^a^ | ***p*-value** | |
|  | (n=4) | (n=84) | (n=11) | (n=5) | (n=19) | (n=32) | (n=41) | (n=44) | (n=12) | (n=13) | (n=63) | (n=12) | (n=152) |  |  |
| Gender  (male, number (%)) | 3  (75) | 31  (37.4) | 6  (54.6) | 4  (80) | 5  (26.3) | 9  (28.1) | 15  (36.6) | 20  (45.5) | 8  (66.7) | 3  (23.1) | 16  (25.4) | 6  (50) | 61  (40.13) | 0.0395 | |
| Age (years),  mean (SD) | 74.55  (11.1) | 64.61  (15.7) | 64.29  (15.5) | 61.4  (25.4) | 61.26  (16.4) | 49.68  (23.4) | 54.40  (24.2) | 61.56  (19.8) | 56.26  (27) | 64.27  (8.53) | 58.85  (19.3) | 62.78  (19.2) | 59.24  (22.6) | 0.076 | |
| Carbapenem-resistant (number (%)) | 4  (100) | 68  (81) | 9  (81.8) | 4  (80) | 10  (52.6) | 25  (78.1) | 1  (2.44) | 38  (86.4) | 2  (16.7) | 1  (7.69) | 54  (85.7) | 8  (66.67) | 16  (10.53) | <.0001 | |
| Hospital  (CGMH-LK, number (%)) | 2  (50) | 69  (82.1) | 10  (90.9) | 3  (60) | 17  (89.5) | 24  (75) | 35  (85.4) | 37  (84.1) | 8  (66.7) | 6  (46.2) | 55  (87.3) | 10  (83.3) | 122  (80.2) | 0.075 | |

^a^: non-KL1/2/3/6/9/10/14/22/47/49/52/81 (non-M1/2/3)

Abbreviation: CGMH-LK, Chang Gung Memorial Hospital-Lin Kou branch

Note: 492 isolates are included in this table.

**Table S6** The distribution of KL types by nosocomial infection and community-acquired infection (*p*-value = 0.015 for Fisher exact test)

|  | **Nosocomial** | **Community** |
| --- | --- | --- |
|  | (N=413) | (N=75) |
| **KL type** | n (%) | n (%) |
| KL1 | 4 (0.97) | 0 (0) |
| KL2 | 69 (16.71) | 12 (16) |
| KL3 | 9 (2.18) | 2 (2.67) |
| KL6 | 2 (0.48) | 3 (4) |
| KL9 | 16 (3.87) | 3 (4) |
| KL10 | 30 (7.26) | 2 (2.67) |
| KL14 | 37 (8.96) | 4 (5.33) |
| KL22 | 42 (10.17) | 2 (2.67) |
| KL47 | 11 (2.66) | 1 (1.33) |
| KL49 | 6 (1.45) | 7 (9.33) |
| KL52 | 58 (14.04) | 4 (5.33) |
| KL81 | 10 (2.42) | 2 (2.67) |
| Other^*^ | 119 (28.81) | 33 (44) |

^*^: non-KL1/2/3/6/9/10/14/22/47/49/52/81 (non-M1/2/3)

Note: Four patients with missing clinical records needed to determine whether the infection was nosocomial or community-acquired were excluded, bringing the total number of isolates included in this table to 488.

**Table S7** The distribution of ST types by nosocomial infection and community-acquired infection (*p*-value = 0.0035 for Fisher exact test)

|  | **Nosocomial (N=221)** | **Community (N=40)** |
| --- | --- | --- |
| **ST type** | **n (%)** | **n (%)** |
| 2 | 100(45.25) | 11(27.5) |
| 10 | 1(0.45) | 5(12.5) |
| 40 | 5(2.26) | 0(0) |
| 57 | 3(1.36) | 1(2.5) |
| 129 | 20(9.05) | 3(7.5) |
| 132 | 3(1.36) | 0(0) |
| 150 | 3(1.36) | 1(2.5) |
| 195 | 2(0.9) | 1(2.5) |
| 221 | 4(1.81) | 0(0) |
| 374 | 2(0.9) | 1(2.5) |
| 1500 | 17(7.69) | 0(0) |
| 1830 | 2(0.9) | 1(2.5) |
| New^a^ | 10(4.52) | 2(5) |
| Other^b^ | 49(22.17) | 14(35) |

^a^: unidentified ST types using the Pasteur database are designated as new.

^b^: Strains with an identified ST type but fewer than two isolates were grouped as ‘other’.

Note: 261 isolates with identified ST types and complete administration date records are included in this table.

In this table, 262 strains subjected to ST typing are included, with one strain excluded due to missing clinical records needed to determine whether the infection was nosocomial or community-acquired, bringing the total number of isolates included to 261.

**Table S8** Carbapenem susceptibility, age, gender, and hospitals (Linkou-CGMH and Kaohsiung-CGMH) for nosocomial and community-acquired infections (*p*-value for Fisher exact test or ANOVA test)

|  | **Nosocomial** | **Community** |  |
| --- | --- | --- | --- |
|  | (n = 413) | (n =75) | *p*-value |
|  |  |  |  |
| Carbapenem susceptibility  (CR, number, (%)) | 213 (48.80) | 27 (35.53) | 0.0018 |
| Age (mean, SD) | 58.68(20.54) | 65.63(20.76) | 0.0074 |
| Gender (Male, number (%)) | 156 (37.50) | 31 (41.33) | 0.9425 |
| Hospital  (CGMH-LK, number (%)) | 340(82.32) | 54(72) | 0.0547 |

Abbreviation: CR, carbapenem resistance; CGMH-LK, Chang Gung Memorial Hospital-Lin Kou branch

Note: Four patients with missing clinical records needed to determine whether the infection was nosocomial or community-acquired were excluded, bringing the total number of isolates included in this table to 488.

**Table S9** Antimicrobial resistance phenotype and carriage of antimicrobial resistance genes in *A. seifertti* AS39

|  | **Antimicrobial resistance phenotype by class/carriage of resistance genes** | | | | |
| --- | --- | --- | --- | --- | --- |
|  | **Carbapenems** | **Cephalosporins** | **Aminoglycosides** | **Tetracyclines** | **Lipopeptides** |
|  | IMP^R^, MER^R^ | CTX^R^, CZF^R^, TAZ^R^, FEP^R^ | GEN^R^ AMI^R^ | TGC^S^ | COL^S^ |
| **Chromosome** | ND | *bla*_ADC-5_ |  | ND | *ept*A |
| **Plasmid (pAS39-2)** | *bla*_NDM-1_ |  | *aph(3')-*VI |  |  |

IMP, imipenem; MER, meropenem; CTX, ceftriaxone; CZF, cefazolin; TAZ, ceftazidime; FEP, cefepime; GEN, gentamicin; AMI, amikacin; TGC, Tigecycline, COL, colistin

ND, not determined; R, resistance; S, susceptible.
